# Stromal NRG1 in luminal breast cancer defines pro-fibrotic and migratory cancer-associated fibroblasts

**DOI:** 10.1038/s41388-021-01719-3

**Published:** 2021-03-10

**Authors:** Mireia Berdiel-Acer, Ana Maia, Zhivka Hristova, Simone Borgoni, Martina Vetter, Sara Burmester, Corinna Becki, Birgitta Michels, Khalid Abnaof, Ilona Binenbaum, Daniel Bethmann, Aristotelis Chatziioannou, Max Hasmann, Christoph Thomssen, Elisa Espinet, Stefan Wiemann

**Affiliations:** 1 Division of Molecular Genome Analysis, German Cancer Research Center (DKFZ), Heidelberg, Germany; 2 Faculty of Biosciences, Ruprecht-Karls-University, Heidelberg, Germany; 3 Department of Gynecology, Martin-Luther-University Halle-Wittenberg, Halle (Saale), Germany; 4 Division of Medical Informatics for Translational Oncology, German Cancer Research Center (DKFZ), Heidelberg, Germany; 5 Department of Biology, University of Patras, Patras, Greece; 6 Institute of Chemical Biology, National Hellenic Research Foundation, Athens, Greece; 7 Institute of Pathology Martin-Luther-University Halle-Wittenberg, Halle (Saale), Germany; 8 e-NIOS PC, Kallithea-Athens, Greece; 9 Roche Diagnostics, Penzberg, Germany; 10 Divison of Stem Cells and Cancer, German Cancer Research Center (DKFZ), Heidelberg, Germany; 11 Heidelberg Institute for Stem Cell Technology and Experimental Medicine (HI-STEM), Heidelberg, Germany

**Keywords:** Breast cancer, Cancer microenvironment, Proteomics, Prognostic markers

## Abstract

HER3 is highly expressed in luminal breast cancer subtypes. Its activation by NRG1 promotes activation of AKT and ERK1/2, contributing to tumour progression and therapy resistance. HER3-targeting agents that block this activation, are currently under phase 1/2 clinical studies, and although they have shown favorable tolerability, their activity as a single agent has proven to be limited. Here we show that phosphorylation and activation of HER3 in luminal breast cancer cells occurs in a paracrine manner and is mediated by NRG1 expressed by cancer-associated fibroblasts (CAFs). Moreover, we uncover a HER3-independent NRG1 signaling in CAFs that results in the induction of a strong migratory and pro-fibrotic phenotype, describing a subtype of CAFs with elevated expression of NRG1 and an associated transcriptomic profile that determines their functional properties. Finally, we identified Hyaluronan Synthase 2 *(HAS2)*, a targetable molecule strongly correlated with *NRG1*, as an attractive player supporting NRG1 signaling in CAFs.

## Introduction

Breast cancer is the leading cause of cancer-related mortality worldwide in females [1]. It is considered a heterogeneous disease that comprises several molecular subtypes based on gene expression analysis or biomarker expression [2, 3]. The family of human epidermal growth factor receptor (HER) of tyrosine kinases (TK) has four members, HER1/EGFR, HER2, HER3 and HER4, and eleven ligands [4]. Overexpression of HER family members favors cancer development, however, it also renders these tumours suitable targets for efficient anticancer therapies [5]. For instance, monoclonal antibodies (mAbs) such as trastuzumab and pertuzumab are usually employed in HER2 overexpressing subtypes [6, 7].

HER3 is emerging as an important component in the luminal subtype of breast cancer, which accounts for about 65–70% of all breast tumours [8]. In agreement with the observation that HER3 is required for cell survival in the luminal but not the basal mammary epithelium [9], luminal breast tumours present the highest levels of *HER3* mRNA [10, 11]. HER3 has weak intrinsic TK activity and needs to form heterodimers with kinase-proficient receptor TKs to be functional [12]. For HER3-positive solid tumours, several HER3-targeting agents have been undergoing clinical evaluation for the last 10 years and currently thirteen mAbs are in phase 1 or 2 clinical studies. In contrast to HER2 inhibitors, HER3 binding antibodies such as lumretuzumab have shown limited clinical efficacy as single agents, but favorable tolerability [13, 14].

The major activating ligand of HER3 is neuregulin 1 (NRG1). Neuregulins (NRGs) are a family of the Epidermal Growth Factor (EGF) ligands that are widely expresed in solid tumors. Most isoforms are synthetized as a transmembrane pro-proteins that undergo proteolity cleavage liberating the EGF-like domain in the extracellular space [15]. In the presence of NRG1, HER3 heterodimerizes mainly with HER2, but also with EGFR or HER4 [16, 17]. These partner molecules induce HER3 tyrosine phosphorylation, binding of adapter molecules and thereby enabling downstream oncogenic signaling via PI3K/AKT, but also MAPK and JAK/STAT pathways. This ultimately leads to tumour progression [17, 18].

Several lines of evidence indicate that NRG1 contributes to the development and progression of different tumour types and its expression has been correlated with poor prognosis in breast cancer, head and neck squamous cell carcinoma and pancreatic cancer [19–22]. The fact that NRG1 is the main activating ligand of HER3, suggests that tumours with high levels of NRG1 could respond better to anti-HER3 targeted therapies [23–25]. Indeed, NRG1-autocrine signaling has been described in a subset of human cancers, such as head and neck and melanoma, to predict sensitivity to HER2/HER3 kinase inhibition [26, 27]. In the case of breast cancer, the relevance of NRG1 ligand in mediating resistance has been previously described [28]. However, in comparison to other cancer entities, the expression of NRG1 in breast tumour cells is usually low and the gene is frequently silenced by DNA methylation [29]. This suggests that an autocrine signaling is unlikely in breast cancer and rather the activation of HER3 in luminal cancer cells might be dependent on NRG1 expressed by cells in the tumour microenvironment.

The tumour microenvironment is typically composed mainly of cancer-associated fibroblasts (CAFs) acompained by immune cells, vascular cells and extracellular matrix (ECM) [30]. CAFs are characterized by the expression of activation markers such as αSMA (alpha smooth muscle actin), FAP (fibroblast activation protein) and FSP1 (fibroblast-specific protein 1) [31], and are a known source of ECM and soluble factors (e.g. growth and inflammatory factors) which impact tumour growth and progression. The potential of CAFs as therapeutic targets or prognostic biomarkers is still under debate, as CAFs appear to represent a heterogeneous group of cells with diverse and even opposing functions that differentially determine tumour fate [32–34].

Here, we study CAF heterogeneity in luminal breast cancer both at the molecular and functional level. Using primary CAFs derived from tumour tissue of luminal breast cancer patients, we demonstrate how heterogeneous expression of NRG1 in CAFs determines response of cancer cells to therapies blocking the HER3 signaling pathway [35, 36]. In addition, we uncover an HER3-independent role of NRG1 associated with migration and proliferation of CAFs, and identified a *NRG1*-correlating transcriptomic network enriched in motility and fibrosis present in CAFs. Finally, we reveal Hyaluronan Synthase 2 (*HAS2*), a targetable molecule, as a supporting player strongly correlating with *NRG1* expression in primary fibroblasts and patient data.

## Results

### *NRG1* is expressed in the stromal compartment of luminal breast cancer

**Fig. 1:**
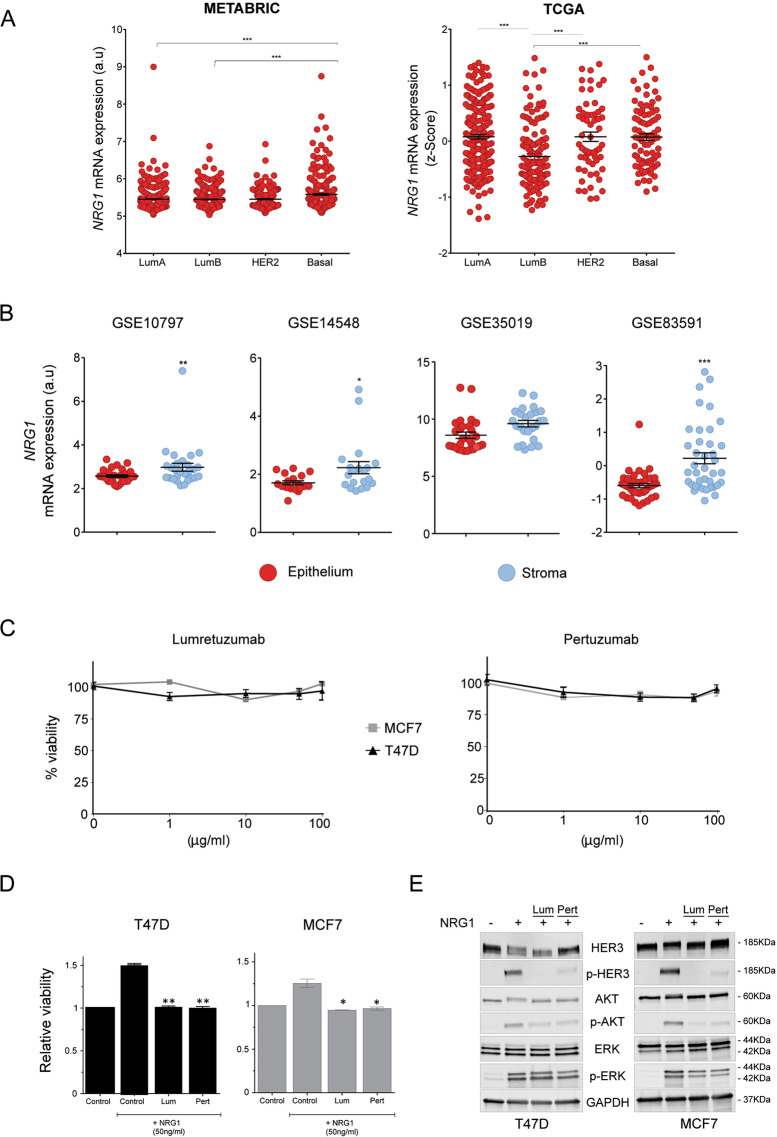
*NRG1* is mostly expressed in the stromal compartment of luminal breast cancer. **A** Dot plots representing expression of *NRG1* in breast cancer subtypes (PAM50) extracted from METABRIC [37] and TCGA [38] datasets. Mean + /− s.e.m represented. Only statistically significant comparisons with luminal subtypes are depicted. ANOVA multiple comparison test (***P < 0.001). **B** Dot plots representing expression of *NRG1* in the epithelial and stromal compartment in four laser capture microdissected (LCM) breast cancer datasets (GSE10797, *n* = 28; GSE14548, *n* = 14; GSE35019, *n* = 53; and GSE83591, *n* = 39). Mean + /− s.e.m represented. Two-tailed paired Student’s *t*-test (*P < 0.05; **P < 0.01; ***P < 0.001). **C** Viability of T47D (black) and MCF7 (grey) measured 72 h after treatment with HER3 mAb lumretuzumab or pertuzumab at indicated doses. Values represent median of three independent experiments (*n* = 5 technical replicates). *U*-Mann–Whitney two-tailed test was applied. No significant differences were observed. **D** Ectopic NRG-1β (50 ng/mL) was added to T47D and MCF7 cell lines and viability quantified. Cells treated with DMEM–F12 media containing 1% FCS were used as a control. Results conditions where cells had been preincubated for 1 h with lumretuzumab (Lum) or pertuzumab (Pert) at 10 µg/mL were normalized to the control condition without NRG1. Two-tailed unpaired Student’s *t*-test (*P < 0.05; **P < 0.01) comparing each treatment with untreated condition (with NRG1). Bars represent average of two independent experiments + /− s.e.m. **E** Western blot showing HER3 and downstream effectors AKT and ERK1/2 (total and phosphorylated levels), 5 min after addition of NRG-1β (50 ng/ml). Some samples were either pre-incubated with mAbs lumretuzumab (Lum) or pertuzumab (Pert) at 10 µg/ml for 1 h. Images are representative of three biological replicates.

To verify the expression pattern of *HER3* in different breast cancer subtypes, we used the public METABRIC [37] and TCGA [38] gene expression datasets. In accordance with previous reports [9], *HER3* showed consistent higher expression in the luminal subtypes in both datasets (Fig. S1A). Conversely, the expression of its main ligand *NRG1* was overall lower with higher levels in basal-like subtypes (Fig. 1A).

Gene expression analysis of bulk tissues comprises mixed signals from different cellular components, masking the contribution of different tumour compartments. Thus, we next explored *NRG1* expression in a collection of breast cancer datasets generated by laser capture microdissection (LCM) of the stromal and epithelial compartments (GSE10797; [39], GSE14548; [40], GSE35019 [41] and GSE83591 [42]). In all LCM datasets explored, expression of *NRG1* was higher in the stromal compartment (Fig. 1B). This indicates that the stromal cells are the major contributors of *NRG1* expression in breast tumour tissue and suggests that activation of the HER3 pathway in tumour cells preferentially happens in a paracrine manner.

In order to define a proper in vitro system for subsequent studies, we analyzed different breast cancer cell lines for expression of the HER family receptors (*EGFR, HER2, HER3* and *HER4)*. As in the primary tissue datasets, cancer cell lines from luminal subtypes (T47D, MCF7 and BT474) showed elevated levels of *HER3* (Fig. S1B).

We focused on luminal A cell lines T47D and MCF7 to avoid masking of HER3 mediated effects by HER2 overexpression (BT474). To test if luminal A cancer cell lines might be intrinsically addicted to HER3 oncogenic signaling [43], cells were challenged with increasing doses of the therapeutic monoclonal antibody lumretuzumab, which blocks binding of NRG1 to HER3 [44], or pertuzumab, which blocks HER2/HER3 heterodimer formation [45]. After 3 days of treatment, viability of cancer cell lines was not affected by HER3 blockage, suggesting no autocrine activation of the HER3 pathway in the luminal A cell lines (Fig. 1C). However, HER3 might still be relevant via paracrine activation. To test this, we added ectopic NRG1 to cancer cells that had or had not been pre-incubated with either lumretuzumab or pertuzumab. Whereas the viability of control cells without antibody-incubation was indeed increased by ectopic NRG1, the effect was abolished by pre-treatment with lumretuzumab or pertuzumab (Fig. 1D). In addition, phosphorylation of HER3 and of its main downstream effectors AKT and ERK was efficiently induced by NRG1 in control cells, while this was strongly prevented upon pre-treatment with lumretuzumab or pertuzumab (Fig. 1E and S1C, D). Together, these data demonstrate that NRG1 activates HER3 pathway via binding to HER3 in a paracrine manner and that this paracrine activation can be blocked with monoclonal antibodies.

### Primary breast cancer-associated fibroblasts express variable levels of NRG1

Cancer-associated fibroblasts (CAFs) are particularly abundant in the stroma of solid tumours [46]. As we had observed that *NRG1* is highly expressed in the stromal compartment of breast tumour tissue (Fig. 1B), we next aimed to determine if CAFs are a source of NRG1. To this end, we established primary cultures of CAFs derived from tumour tissues from six breast cancer patients clinically classified as luminal subtype (Table S1).

**Fig. 2:**
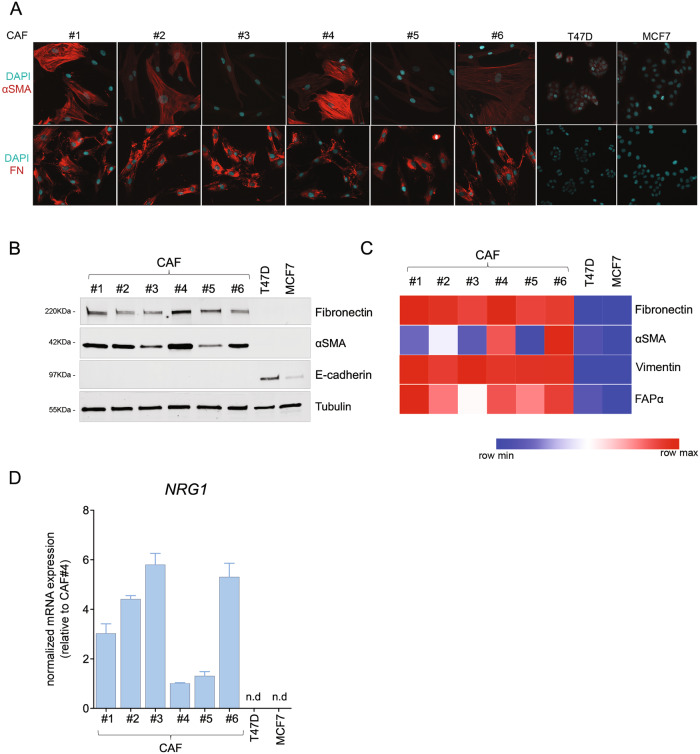
Primary breast cancer-associated fibroblasts express variable levels of *NRG1*. **A**, **B** Immunofluorescence (**A**) and western blot (**B**) of common activation markers αSMA (red, upper panel) and fibronectin (FN1) (red, lower panel) in CAFs and luminal cancer cells lines (T47D and MCF7) under study. In (**A**), nuclei counterstaining with DAPI (blue). Representative images are shown. In (**B**), the epithelial marker E-cadherin served as marker for epithelial cells. Tubulin was used as a loading control. **C** Heatmap representing protein values of common mesenchymal markers αSMA, fibronectin, vimentin and FAPα in all six CAFs under study and in two luminal breast cancer cell lines obtained by reverse phase protein array (RPPA). Color intensities are ranked per each antibody (red = maximum, blue = minimum). **D** Expression of *NRG1* transcript in all six CAFs and in luminal breast cancer cell lines (T47D and MCF7). Values were normalized to the geometric mean of *ACTB* and *GAPDH*, and are shown as relative to CAF#4. Bars represent mean + /− s.e.m of two independent experiments.

The isolated cells showed the characteristic fibroblast morphology as well as expression of common CAF activation markers such as αSMA, fibronectin (FN1) and FAP (Fig. 2A–C and S2A). We further confirmed their CAFs lineage by comparing RNA seq expression data of specific markers in CAFs vs cancer cell lines (Table S2).

Next, we analysed mRNA transcript and protein expression levels of *NRG1* in the CAF lines and in the two luminal breast cancer cell lines. In line with the LCM patient data, *NRG1* was expressed by all CAF lines, while no expression could be detected in cancer cells (Fig. 2D and S2B). Interestingly, NRG1 levels were heterogeneous among the different fibroblasts despite having been isolated from tumours of the same subtype. Collectively, these results demonstrate that NRG1 is expressed by CAFs in the stroma of luminal breast cancer patients and reinforce the concept of a paracrine-driven activation of HER3 in the luminal breast cancer subtype.

### Different levels of NRG1 secreted by CAFs determine activation of HER3 in cancer cells

To ascertain whether different expression of NRG1 by CAFs translates into variable activation of the HER3 pathway in cancer cells, we stimulated T47D and MCF7 cancer cells with conditioned media (CM) from the isolated CAFs, and used lumretuzumab to block ligand–receptor binding.

**Fig. 3:**
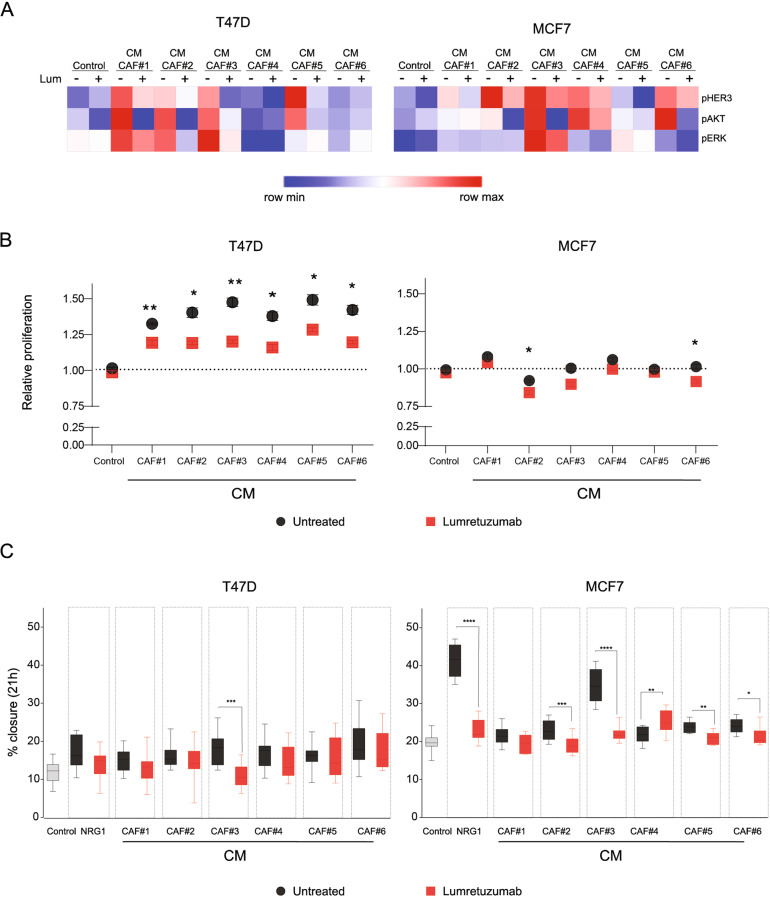
Activation of HER3 in cancer cells by secreted NRG1 is CAF-dependent. **A** Heatmap representing relative phosphorylation levels of HER3, AKT and ERK1/2 in T47D and MCF7 cancer cells, quantified by RPPA. CAF conditioned media (CM) was added to cancer cells for 5 min that had or had not been pre-incubated with lumretuzumab (Lum) (10 µg/ml) for 1 h. All CM were obtained with 1% FCS, and DMEM-F12 with 1% FCS was used as control. Values represent median of three technical replicates and three biological replicates. Color intensities are ranked per each antibody (red = maximum, blue = minimum). **B** Relative proliferation of T47D and MCF7 cancer cells after 72 h with different CAF-CM with lumretuzumab (red squares) or untreated (black circles), compared with cells cultured in control media (DMEM-F12, 1% FCS) and untreated. Dots/squares represent mean + /− s.e.m of three independent biological replicates (*n* = 4). *P* values were determined by two-tailed *U*-Mann–Whitney test for each CM (*P < 0.01; **P < 0.001). **C** Percentage of closure in a scratch assay of T47D or MCF7 cancer cells, after 21 h of treatment with 10 μg/ml lumretuzumab (red) or untreated (black), and with conditioned media (CM) of indicated CAFs. DMEM-F12 1% FCS was used as negative control and NRG-1β (50 ng/ml) as positive control. Box plots correspond to the mean and + /− s.e.m. of two independent experiments (each with *n* = 6 technical replicates). Two-tailed *U*-Mann–Whitney test for each CM (*P < 0.01; **P < 0.001; ***P < 0.0005; ****P < 0.0001).

In order to detect phosphorylation levels of HER3 and its main downstream effectors AKT and ERK in a sensitive and quantitative manner, we applied Reverse Phase Protein Array (RPPA) technology [47]. Incubation with ectopic NRG1 as well as blockage with lumretuzumab were used as positive controls (Fig. S3A). Phosphorylation of HER3 observed in cancer cells upon stimulation with the different CM was CAF- and cancer cell-dependent, achieving different phosphorylation levels (Fig. 3A, red = maximum, blue = minimum). In both cell lines we observed variable levels of phosphorylation of the HER3 pathway induced by CM from the different CAFs, which was specially stronger for CAF#3, the one that showed higher expression of *NRG1* (Fig. 2D and Fig. S2B). Pre-incubation with lumretuzumab reduced phosphorylation of HER3 and its effectors in all conditions, confirming a HER3 activation mediated by the NRG1-present in the CAF-CM.

Next, we tested the ability of NRG1 in the CAF-CM to promote proliferation of cancer cells. The proliferation rate of cancer cells did not follow the same trend as *NRG1* expression; however, blockage of NRG1-HER3 signaling by lumretuzumab, decreased proliferation (Fig. 3B) of T47D cancer cells while a minor effect was observed for MCF7 cells.

Due to the established role of *NRG1* in epithelial to mesenchymal transition (EMT) and migration processes [48], we next measured if also migration abilities of the cancer cells were altered in presence of CAF-CM and if these were dependent on HER3 activation by NRG1. Consistently, CAF-CM increased migration of MCF7 cancer cells mainly, and the effect was diminished with lumretuzumab whereas a less prominent effect was obtained for T47D cancer cells. (Fig. 3C). The strongest effect was observed for CAF#3-CM, the CAF culture that expressed the highest level of NRG1.Treament with lumretuzumab of cancer cells cultured in control media, did not alter their migration abilities, confirming the absence of an autocrine HER3 activation (Fig. S3B). While the T47D cell line showed a boosting effect in proliferation but more modest migration phenotype, the opposite occurred for the MCF7 cell line. The latter showed little or non-increase in proliferation while stronger effects were observed for migration, which could be explained by the molecular switch from proliferative to migratory phenotype, where cells reduce their proliferation rate in order to enhance their migratory potential.

Taken together, these results indicate that CAFs isolated from tumour tissue of luminal breast cancer specimens differently activate the HER3 pathway and regulate proliferation and/or migration of cancer cells via NRG1 secretion.

### Heterogeneous expression of *NRG1* among CAFs

**Fig. 4:**
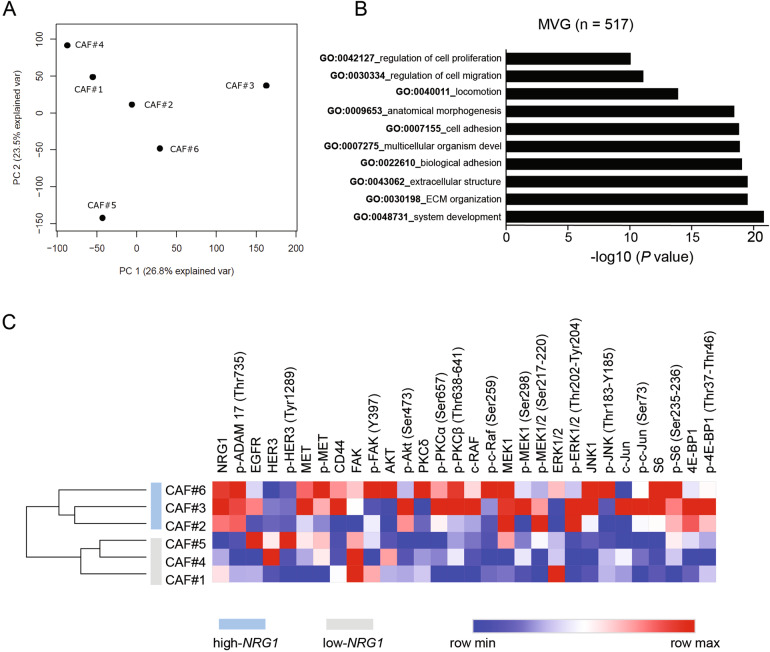
Heterogeneous expression of *NRG1* among CAFs. **A** Principal component analysis PC1 and PC2 of all six CAF lines under study. **B** Biological processes (gene ontology) enriched in CAFs most variable genes (MVG). Graph bars represent adjusted *P* value. **C** Proteomic profile of CAFs based on reverse phase protein array (RPPA). Intensity values (red = maximum, blue = minimum) are ranked per each antibody to compare between samples. Values represent median of three technical replicates. Unsupervised clustering by Euclidian distance separates CAFs (high-NRG1, blue and low-NRG1, grey) respectively.

Despite all different cultures of CAFs were derived from luminal breast cancer tissue, they showed variable capacities to activate the HER3 pathway in luminal breast cancer cells via NRG1 (Fig. 3A–C). To investigate the possible differences between the isolated CAFs in a global approach, we performed RNA sequencing of the six primary CAF lines. Principal component analysis (PCA) revealed that gene expression among fibroblasts was indeed scattering (Fig. 4A). To elucidate which genes were contributing to this variance, we analyzed the most significant variable genes (MVG) amongst the different CAF lines. A list of 517 significant genes was defined, with *NRG1* ranked among them (Table S3). Other genes listed as highly heterogeneous were *ACTA2* and *S100A4* coding for αSMA and FSP1 respectively, two well accepted CAF markers, although not correlated with *NRG1* expression (Fig. S4A).

Next, we performed functional analysis using gene ontology (GO) terms in the Bioinfominer online platform (https://bioinfominer.com) [49] for the 517 MVG. Functional categories related to extracellular matrix, cell adhesion and locomotion were significantly enriched (Fig. 4B and Table S4). Among the functional categories that were differing in fibroblasts, we found regulation of proliferation. In line with this, nuclei counting of fibroblasts along several days revealed a heterogeneous proliferation degree among the CAFs lines (Fig. S4B).

To comprehend upto which extent *NRG1* contributed to fibroblasts variability, we split CAFs based on *NRG1* expression into low-*NRG1* (lower than mean: CAF#1, CAF#4, CAF#5) and high-*NRG1* (higher than mean: CAF#2, CAF#3, CAF#6) CAFs (Fig. S4C). We perfomed targeted proteomic analysis for the CAFs under study to elucidate if phosphorylation status of effectors of the HER pathway (e.g. ERK1/2, AKT, MET, S6K, ADAM17 [50]) were differing among the lines. Unsupervised clustering grouped CAFs into two groups demonstrating different activation of the HER3 pathway. Interestingly, NRG1 expression was sufficient to separate those CAFs (Fig. 4C). Moreover, analysis of the transcription factor genes enriched in high-*NRG1* CAFs disclosed c-JUN as the main transcription factor regulating high-*NRG1* transcriptome (Fig. S4D and Table S4 Table S5). Strikingly, phosphorylation of the transcription factor c-JUN, described as a central molecular mediator in fibrotic conditions [51] and hyperactivated in high density stroma breast cancer tissue [52], was higher in high-*NRG1* CAFs (CAF#2, CAF#3, CAF#6).

Collectively, these results underline the relevance of NRG1 as a heterogeneous factor expressed by CAFs in luminal breast tumours.

### *NRG1*-associated transcriptome correlates with migration processes in CAFs

**Fig. 5:**
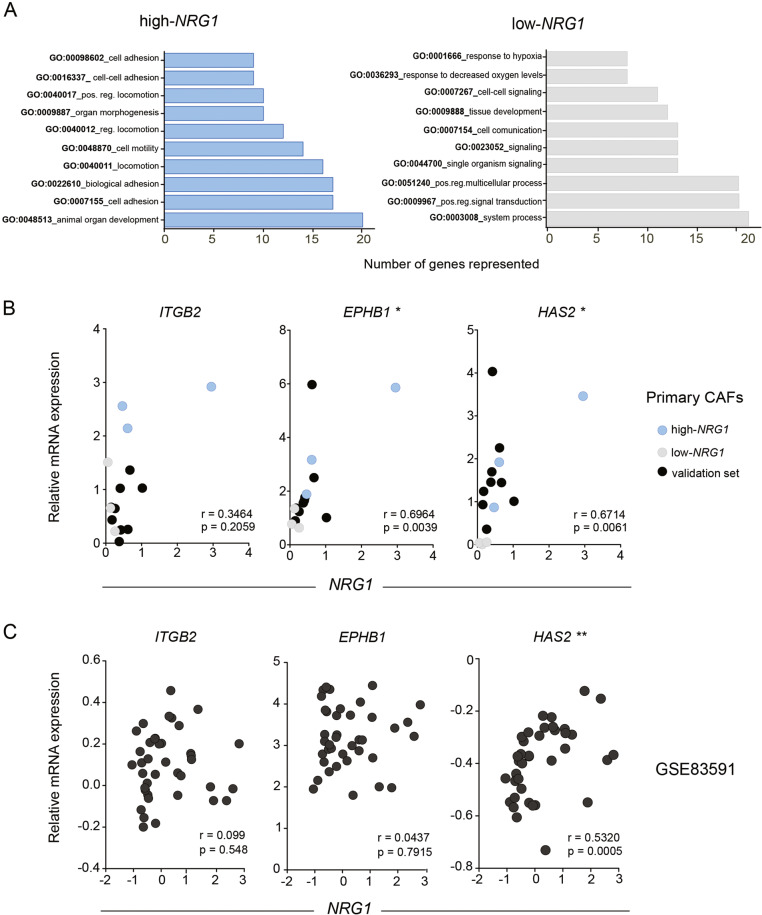
Genes associated to *NRG1* expression. **A** Functional classification of genes by gene ontology (GO). Biological processes represented in each group of CAFs. Graph bars showing number of genes with significant *P* value for high- (blue) and low- (grey) NRG1 CAFs respectively. **B** Expression correlation of *NRG1* and candidate genes quantified by RT-PCR in primary CAFs from two independent sources, six lines from Halle University (discovery set, blue = high-*NRG1* and grey = low-*NRG1*) and another nine from Breast Cancer Now (validation set, black). Each dot represents the average of >3 independent experiments. Spearman *r* correlation and two-tailed *P* value are indicated. **C** Expression correlation of *NRG1* and indicated genes in the stroma compartment of the LCM dataset GSE83591 (*n* = 39). Spearman correlation *r* and *P* values are indicated.

We next wanted to elucidate if different expression of *NRG1* in fibroblasts was associated with specific transcriptional programs. To this end, we performed differential expression analysis between the designated high and low-*NRG1* groups of CAFs. A total of 102 genes were upregulated and 151 were downregulated in the high- vs low-*NRG1* CAFs (adj *P* value < 0.05 and absolute logFC > 0.5) (Table S6). Gene Ontology (GO) analysis revealed that genes enriched in the high-*NRG1* CAF group were mainly related with adhesion and motility processes. In contrast, terms enriched in low-*NRG1* CAFs were associated to signaling and metabolic processes (Fig. 5A). Based on the list of significant differentially expressed genes (Table S6), we used BioinfoMiner (https://bioinfominer.com) to explore systemic processes and driver genes characteristic of each group. In line with the GO results (Fig. 5A), driver genes (*P* value < 0.002 and log2FC > 2) in high-*NRG1* CAFs included *ITGB2*, *EPHB1* and *HAS2*, known locomotion and extracellular matrix reorganization related genes. In contrast, driver genes in low-*NRG1* CAF included genes such as *PTGIS, TRH, WNT2* or *JAG1*, involved in cell signaling and metabolic processes (Table 1).

**Table 1:** Priorization list of driver genes in each group of CAFs.

High-*NRG1*			Low-*NRG1*		
Gene	Log2FC (fold change)	*P* value	Gene	Log2FC (fold change)	*P* value
*WT1*	2.47	1.90E–03	PTGIS	−3.3	9.26E–23
ITGB2	2.32	2.48E–06	ERBB3	−2.78	1.18E–04
EPHB1	2.02	3.41E–09	CRLFH1	−2.77	9.00E–06
HAS2	2.02	3.69E–03	TRH	−2.38	3.31E–03
S1PR1	1.91	6.58E–03	WNT2	−2.23	4.30E–03
EFNB2	1.91	3.80E–02	JAG1	−2.17	4.27E–03
WNT5A	1.89	4.29E–04	IL18	−2.15	1.20E–02
WNT7B	1.88	4.40E–02	PLCB1	−2.04	1.14E–11
NRG1	1.72	2.01E–06	CD34	−1.96	3.20E–02
GAS6	1.64	7.14E–03	F2R	−1.95	7.14E–03
COL6A3	1.23	1.35E–03	SGCG	−1.78	7.40E–03
TYRO3	1.09	1.80E–02	FZD4	−1.73	5.00E–02
PDGFC	1.03	3.00E–02	WNT2B	−1.7	2.00E–02
BVES	1.02	4.63E–03	ABCA7	−1.48	1.25E–03
ETS1	0.9	2.00E–02	JAK3	−1.45	7.95E–03
			HES1	−1.33	1.90E–02
			HIF1A	−0.85	1.30E–02

Analysis of DEG using BioInfoMiner (https://bioinfominer.com) identifed a list of priorization genes as hub nodes for high-*NRG1* (left panel) and low-*NRG1* CAFs (right panel), respectively. Fifteen genes were significantly upregulated defining high-*NRG1* CAFs, and seventeen genes significantly downregulated in high-*NRG1* CAFs, defining low-*NRG1* CAFs.

Altogether, these results determine *NRG1* as a stromal marker discerning fibroblasts with different transcriptional programs.

### *HAS2* expression correlates with *NRG1* in the tumour stroma of patient samples

In order to obtain a signature of genes linked with *NRG1* expression in tumour stromal fibroblasts, we selected those genes with the strongest correlation with *NRG1* (Pearson *r* > 0.8), and with a log2FC > 2 in high- vs low-*NRG1* CAFs (Fig. S5A and Table S7).

We validated the correlation of these genes (i.e. *ITGB2*, *EPHB1* and *HAS2*) with *NRG1* in a second set of primary CAFs from an independent source (https://breastcancernow.org/breast-cancer-research/breast-cancer-now-tissue-bank) (Fig. 5B), and we explored their expression in LCM stroma datasets from breast cancer patients (Fig. 5C). This analysis confirmed a significant positive correlation between *NRG1* and *HAS2* (Fig. 5C). Next, we checked the expression in the TCGA dataset covering bulk tumour tissue, only considering those samples with tumour purities <50% to select tumours with high stroma content. From the candidates investigated, *HAS2* was significantly correlated with *NRG1* also in TCGA dataset (Fig. S5B).

Taken together, these analyses uncover *HAS2* as a stromal gene highly correlated with *NRG1* in luminal breast cancer patients.

### *NRG1* downregulation in CAFs downregulates *HAS2 and* impairs their migration

**Fig. 6:**
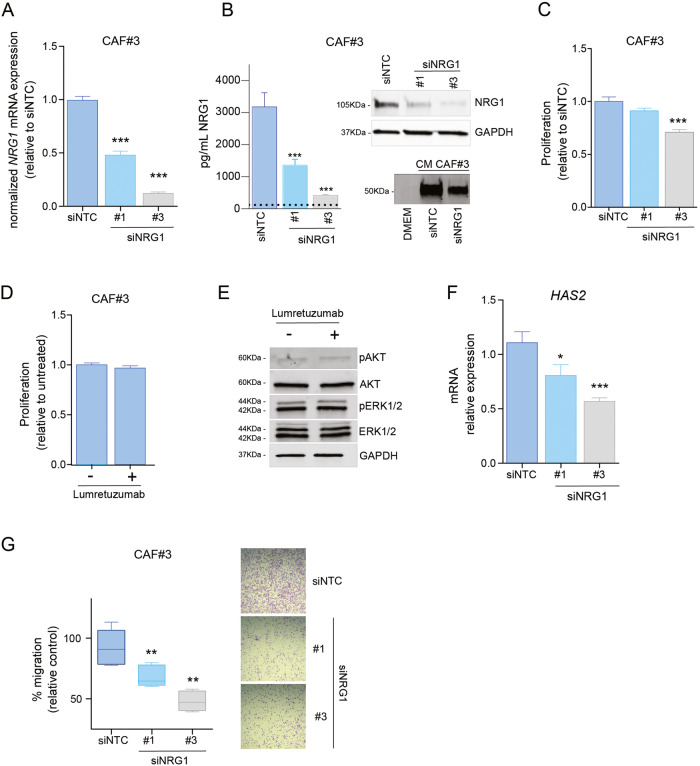
Functional implications of *NRG1* in CAFs. **A** Downregulation of *NRG1* at RNA level (**A**) in CAF#3 (high-*NRG1*) using two independent siRNAs (siNRG1 #1, #3). Bar graphs represent average of three independent experiments and three technical replicates each. Two-tailed *U*-Mann–Whitney test comparing with siRNA non-targeting control (siNTC) (***P < 0.01). **B** Downregulation of NRG at protein level using two independent siRNAs (siNRG1 #1, #3). Left panel shows secreted NRG1 in the conditioned media measured using ELISA assay. Right panel shows western blot of cell-bounded NRG1 (upper) and secreted NRG1 in conditioned media obtained via immunoprecipitation (lower), were concentrated DMEM was used as a negative control. Images are representative of two independent experiments. **C** Relative cell growth of CAF#3 was measured 72 h post re-seeding (5 days post transfection) either with two different siRNAs targeting *NRG1* (siNRG1 #1, #3) or a non-targeting control siRNA (siNTC). *U*-Mann–Whitney test was applied for statistical analysis (***P < 0.01). **D** Relative cell number of CAF#3, 72 h after treatment with 10 μg/ml lumretuzumab (Lum). No statistical differences were obtained. **E** Total protein and phosphoprotein levels for AKT and ERK1/2 in CAF#3 after 24 h of treatment with or without lumretuzumab (Lum) at 10 μg/ml. GAPDH was used as loading control. **F** *HAS2* mRNA levels in cells treated either with a non-targeting control siRNA (siNTC) or two different siRNAs targeting *NRG1* (siNRG1#1, #3) relative to siNTC. Graph bars represent average of three independent experiments (*n* = 3 technical replicates). Two-tailed *U*-Mann–Whitney test comparing with siRNA non-targeting control (siNTC) (*P < 0.05; ***P < 0.001). **G** Migration of CAFs transfected either with a non-targeting control siRNA (siNTC) or two siRNAs targeting NRG1 (siNRG1#1, siNRG1#3) and normalized with seeding control. Whiskers in the box plot represent minimum and maximum values of three independent experiments (six technical replicates each). Two-tailed *U*-Mann–Whitney test comparing with siRNA non-targeting control (siNTC) (**P < 0.01). Crystal violet staining of transwell inserts representing migration after 8 h of CAF#3 transfected either with a control siRNA (siNTC) or either of siRNAs targeting NRG1 (siNRG#1, siNRG1#3).

We had observed that high-*NRG1* CAFs displayed higher proliferation rates and showed signatures of proliferation (Fig. S4B and Table S4). We thus explored the potential contribution of *NRG1* expression to this phenotype in CAFs. To this end, we first knocked down *NRG1* in high-*NRG1* CAF lines by transient transfection. We used two independent siRNA sequences inducing different degrees of downregulation, thus mimicking heterogeneous downregulation of NRG1 at RNA and at protein level, both secreted and cell-bounded (Fig. 6A, B and Fig. S6A). Functional downregulation of *NRG1* was confirmed by using the CAF-siNRG1 CM on cancer cells. Both T47D and MCF7, showed a decrease in HER3 phosphorylation after incubation with CM from siNRG1 transfected CAF#3, which paralleled the extent of *NRG1* downregulation in the CAFs (Fig. S6B). Similarly, migration (Fig. S6C) and proliferation of cancer cells induced by CAFs CM was also diminished upon *NRG1* downregulation in CAF#3. Effects obtained with siNRG1#3 were comparable to those obtained upon lumretuzumab treatment (Fig. S6D). Similar pattern was obtained with the CM of CAF#2 and CAF#6, although showing a less prominent effect (Fig. S6E) probably due to the different basal level of NRG1 in the control condition. Having shown that the levels of *NRG1* downregulation in CAFs were sufficient to affect cancer cells, we next investigated the effect of NRG1 in CAFs. We observed that decreased NRG1 expression resulted also in a reduced proliferation rate in CAFs themselves (Fig. 6C); however, ectopic addition of NRG1 did not rescue that phenotype (Sup Fig. S6F). This indicates that CAF-secreted NRG1 positively contributes to proliferation of cancer cells while it does not affect CAFs. Thus, we next investigated if the proliferative effect of NRG1 expression in CAFs was dependent on the binding of NRG1 to HER3. Contrary to cancer cells (Fig. 3B and Fig. S1D), inhibition of NRG1 binding to HER3 by lumretuzumab, neither decrease proliferation of CAFs nor phosphorylation of AKT and ERK (Fig. 6D and E). Altogether, these data demonstrate that the effect exerted by NRG1 on CAFs proliferation is independent of the canonical binding of secreted NRG1 to HER3. Further supporting this finding, expression levels of HER3 in CAFs were very low compared with expression levels in cancer cells and even lower in high-*NRG1* CAFs (Fig. S6G). Of note, binding of NRG1 to HER4 receptor was not considered due to its undetectable expression in CAFs (Fig. S6H).

Transcriptomic profiling had revealed an enrichment of signatures related to migration in high-*NRG1* CAFs (Fig. 5A). In addition, *NRG1* correlated with *HAS2*, a known mediator of migration, in several CAF lines and patient stroma datasets (Fig. 5B and C). We thus wondered if *NRG1* downregulation could affect also *HAS2* levels and migratory capacity of CAFs. Remarkably, we observed a proportional decrease in *HAS2* mRNA transcript levels upon knockdown of *NRG1* (Fig. 6F) which was associated with a marked decrease of the migration of CAFs (Fig. 6G).

Collectively, this data suggest a HER3-independent role of NRG1 in cancer-associated fibroblasts that modulates their proliferation and migration and that could be driven by its intracellular domain (ICD).

## Discussion

Overexpression of human epidermal growth factor receptor 3 (HER3) plays an important role in cancer development as well as acquired drug resistance in a wide variety of solid tumours [53, 54]. It has been associated with worse clinical outcome, and monoclonal antibodies such as lumretuzumab have been developed to neutralize its activity by blocking the binding of its ligand NRG1 [35, 44]. Several pre-clinical and clinical studies have supported NRG1 as a predictive biomarker for anti-HER3 targeted therapies [13, 23, 55]. NRG1-mediated autocrine signaling in cancer cells has been reported to underlie sensitivity to anti-HER2 therapies in certain ovarian and head and neck tumours [26, 56]. In our study, we show that in luminal breast cancer, the stromal compartment is the major contributor of NRG1 expression and that its expresion is non detectable in cancer cells [29]. Our results suggest that in luminal breast cancer, cancer cells depend on paracrine NRG1 to activate downstream pathways. Here, we used primary fibroblasts isolated from luminal breast cancer tissue and demonstrated that NRG1 produced and secreted by CAFs is sufficient to activate the HER3 pathway in cancer cells. Activation of HER3 by CAF CM promotes phosphorylation of main downstream activators AKT and ERK, leading to proliferation and migration of cancer cells. The use of lumretuzumab, a humanized monoclonal antibody that selectively binds to the extracellular domain of HER3 thereby blocking the binding of NRG1, is able to prevent that phenotype in a NRG1-dependent manner, as similarly occurred when using of CM from NRG1 downregulated CAFs. Thus, we suggest that the utility of NRG1 as a predictive biomarker to anti-HER3 therapies in luminal breast cancer may be provided by the stromal compartment, while analysis of bulk tumour tissues may dilute its detection [57].

It is widely accepted that CAFs are a heterogeneous population of mesenchymal cells defined by their diversity in functions, markers and origins [34]. Several studies have compared gene expression in disease-free fibroblasts and CAFs derived from various tissues to obtain information on stromal pathways facilitating malignant phenotypes [58–60]. Other works have been oriented towards identifying specific lineages within CAFs based on their tumour promoting abilities to identify subpopulations [61–63]. Also, recent studies have described novel approaches for the study of biological function and targeting of CAFs [64]. Here, we have identified heterogeneous expression of *NRG1* in the stroma of luminal breast cancer tissue. Its higher expression defines CAFs with an associated motile, fibrotic transcriptome and phenotype. In addition, unsupervised clustering based on the proteomic profile of relevant signaling effectors such as ERK1/2, AKT,MEK, and ADAM17 (a disintegrin metalloprotease responsible for the cleavage of membrane bound growth factors such as NRG1 [65]), classified CAF lines in the same high- and low-groups, supporting the role of NRG1 in defining a different activation status.

The differential expression analysis conducted in this study revealed 102 genes upregulated in high-*NRG1* CAFs, which were enriched in gene signatures related to a motile phenotype. We identified *ITGB2* and *EPHB1* as strongly correlating with *NRG1* expression in breast CAFs. Indeed, both *ITGB2* and *EPBH1* have been previously documented to play significant roles in polarization and cell migration [66].

**Fig. 7:**
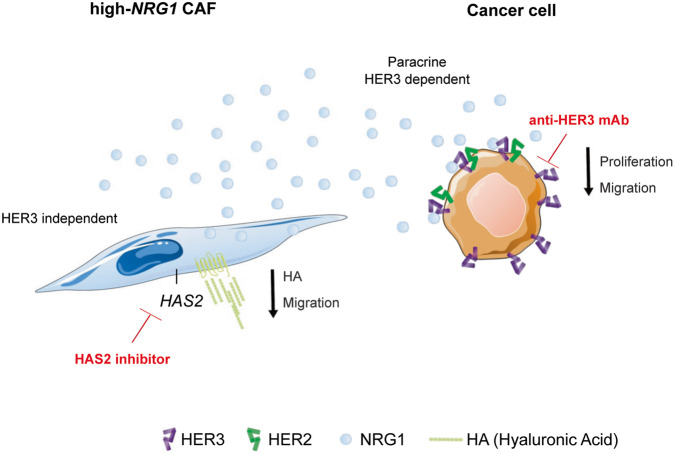
Model for dual targeting of high-*NRG1* stroma in luminal breast cancer. High-*NRG1* CAFs (blue) secrete high quantities of NRG1 (blue circles) promoting paracrine activation of HER3-receptor in cancer cells and thereby inducing proliferation and migration processes. Use of lumretuzumab blocks HER3 receptor in cancer cells and avoids binding of NRG1 thereby reducing proliferation and migration processes in the tumour cells. Suggested NRG1 non-canonical signaling induces proliferation and migration in CAFs, in a HER3-independent manner. CAFs are highly proliferative and migratory also due to increased expression of *HAS2* and secretion of HA. Additional use of a HAS2 inhibitor could help to reduce expression of *HAS2*, and thus secretion of HA reducing migration of CAFs (/https://smart.servier.com/).

Finally, we revealed *HAS2* (Hyaluronan Synthase 2) as a gene that is strongly correlated with *NRG1*, not only in primary CAFs but also in patient stroma datasets. HAS2 is responsible for the synthesis of hyaluronan (HA), a glycosaminoglycan with a demonstrated role in cancer initiation and progression and whose elevated accumulation in either the stroma or tumour parenchyma of many cancers is linked to tumour aggressiveness and poor outcome [67, 68]. We suggest that in our system, correlation of *HAS2* and *NRG1* is consequence of a regulatory mechanism in which NRG1 expressed by CAFs regulates *HAS2* expression and modulates migratory potential of the fibroblasts by NRG1 non-canonical signaling [69, 70]. We hypothesize that this regulation is the result of NRG1 backward signaling, which has been previously described in the development of the nervous system [71]. We suggest that proteolytic cleavage of pro-NRG1 in the intracellular domain induces the release of NRG1 ICD, which triggers backward signaling by nuclear translocation and modulation of gene transcription. Although additional molecular characterization will be necessary to further decipher the exact mechanism of this regulation, the strong correlation observed in tumour stroma of luminal breast cancer patient samples clearly propose these two molecules as potential CAFs biomarkers and therapeutic targets. Thus, we consider that dual targeting of NRG1 and HAS2 may be an interesting treatment strategy applicable for tumours with high expression of NRG1. Indeed, inhibition of *HAS2* upon treatment with its specific inhibitor 4-methylumbelliferone (4-MU, also known as hymecromone), has proven successful in reducing tumour stroma in pancreatic ductal adenocarcinoma (PDAC) [72, 73], which also shows high expression of NRG1 [24]. We hypothesize that for breast cancer, on one side, treatment with anti-HER3 monoclonal antibodies would reduce the proliferation and migration of cancer cells by blocking stromal NRG1 binding, thus diminishing tumour aggressiveness. Concomitantly, the use of a specific HAS2 inhibitor would induce a reduction of the stroma content by decreasing HA synthesis by CAFs (Fig. 7) further contibuting to tumour growth/aggressiveness wane. Notably, both lumretuzumab and 4-MU are approved drugs currently used in several clinical trials. Ultimately, this tailored combination therapy represents a novel treatment approach from which not only luminal breast cancer patients but possibly other tumor entities with high levels of stromal NRG1 could benefit.

## Materials and methods

### Clinical samples, cell isolation and characterization

Tumour tissue was collected from patients (*n* = 6) undergoing surgery for breast carcinoma at the Department of Gynecology, Martin-Luther-University Halle-Wittenberg in Halle (Saale), Germany, following ethical approval by the Ethics committee of Martin-Luther-University Clinics Halle-Wittenberg (Halle Saale), Germany, and written informed consent provided by the patients. Tumour tissue was mechanically minced into pieces (1–4 mm^3^) and centrifuged at 1600 rpm for 10 min. After fat removal, pellet containing small pieces was resupended in DMEM/F12 (10% FCS, 1% P/S and 1% fungizone), filtered with a cell strainer (70 µm) and plated in a 60 mm culture dish. Outgrowth of cells was daily checked and medium renewed twice per week. After complete outgrowth in a 60 cm^2^ dish, cells were passaged with 0.25% Trypsin-EDTA (Gibco, Life Technologies) and fibroblasts seeded into a new 100 mm culture dish. After three cell passages, morphologically homogeneous cultures containing only fibroblasts were obtained and RNA and protein were collected for further characterization. To obtain conditioned media, 2.5 × 10^5^ CAFs were seeded in 100 mm culture dish. Once cells were attached, media was replaced by DMEM/F12 (1% FCS, 1% P/S), incubated for 24 h and collected for their further use.

### Reverse Phase Protein Array (RPPA)

RPPA experiments were performed as previously described [47, 74]. Briefly, cell lysates from three biological replicates for each condition were spotted in nitrocellulose-coated glass slides (Oncyte Avid, Grace-Biolabs, Bend, OR, USA) in technical triplicates. All the primary antibodies used were previously validated through Western blots to test their specificity. Signal intensities of spots were quantified using GenePixPro 5.0 (Molecular Devices, Sunnyvale, CA, USA). Intensity values were log2 transformed and plotted using morpheus software (https://software.broadinstitute.org/morpheus/). List of antibodies used is provided in Table S8

### Drug treatments

Lumretuzumab and pertuzumab were provided by Roche Diagnostics GmbH (Penzberg). Prior to addition of CAF-CM or human recombinant NRG-1β (4711, BioCat), cells were pre-treated with either lumretuzumab or pertuzumab (10 μg/ml) for 30 min in low serum media (1% FCS). For viability/proliferation assays, media was removed and CAF-CM or low serum media (with or without 50 ng/ml NRG-1β) was added and incubated for 3 days. For short perturbation assays, incubation time was 5 min prior to lysates collection.

### RNA sequencing and data processing

RNA sequencing of the six CAF lines was performed at the Genomics Core Facility of German Cancer Research Center (DKFZ- https://www.dkfz.de/gpcf) using the Illumina HiSeq 4000 platform and the Illumina stranded TruSeqRNA paired end sequencing kit. After quality control, reads were mapped to the human genome hg38 using STAR (version 2.3.0e), and reverse strand read counts were determined using featureCounts (version 1.5.1). The reads were mapped to ENSEMBL IDs and gene symbols. Exonic gene lengths and TPMs (Transcript Per kilobase Million reads) were calculated in *R*. To avoid infinite values, a count of one was added to each TPM value and the resulting values were then log2 transformed.

### Functional analysis: gene ontology

We used the BioInfoMiner online platform to investigate which biological process Gene Ontology (GO) terms [75, 76] were enriched in the list of differentially expressed or highly variable genes. BioInfoMiner exploits biological hierarchical vocabularies by mapping the genes to a genomic network created from semantic data. It prioritizes them based on the topological properties of the network after minimizing the impact of semantic noise (bias) through different types of statistical correction. It detects and ranks significantly altered processes and the driver genes involved. The BioInfoMiner platform is available online at the website https://bioinfominer.com.

## Supplementary information

Supplementary Material

Figure S1

Figure S2

Figure S3

Figure S4

Figure S5

Figure S6

Table S1

Table S2

Table S3

Table S4

Table S5

Table S6

Table S7

Table S8

## Data Availability

Gene expression data of the primary CAF lines is available in the ArrayExpress data repository at https://www.ebi.ac.uk/arrayexpress/experiments/E-MTAB-10075. All other data generated or analysed during this study are included in this published article [and its supplementary information files].
