# Supplementary material for: Stromal NRG1 in luminal breast cancer defines pro-fibrotic and migratory cancer-associated fibroblasts
Source: Oncogene. 2021 Mar 10;40(15):2651–66. doi: 10.1038/s41388-021-01719-3 (PMC8049869; doi:10.1038/s41388-021-01719-3)
Supplement: Supplementary file 1 — Supplementary Material [file 41388_2021_1719_MOESM1_ESM.docx]

**Stromal NRG1 in luminal breast cancer defines pro-fibrotic and migratory cancer-associated fibroblasts**

**NRG1-specific fibroblasts in luminal breast cancer**

Mireia Berdiel-Acer*^1^, Ana Maia^1,2^, Zhivka Hristova^1,2^, Simone Borgoni^1,2^, Martina Vetter^3^, Sara Burmester^1^, Corinna Becki^1^, Birgitta Michels^1^ Khalid Abnaof^1^, Ilona Binenbaum^4,5,6^, Daniel Bethmann^7^, Aristotelis Chatziioannou^6,8^, Max Hasmann^9^, Christoph Thomssen^3^, Elisa Espinet^10,11^, Stefan Wiemann*^1^

**Supplementary Information**

This supplementary information contains the following:

[Supplementary Figures 2](#_Toc63079312)

[Supplementary Figure Legends 8](#_Toc63079313)

[Legends for Supplementary Tables 12](#_Toc63079314)

[Supplementary Materials and Methods 14](#_Toc63079315)

[Supplemental References 20](#_Toc63079316)

Supplementary Figures


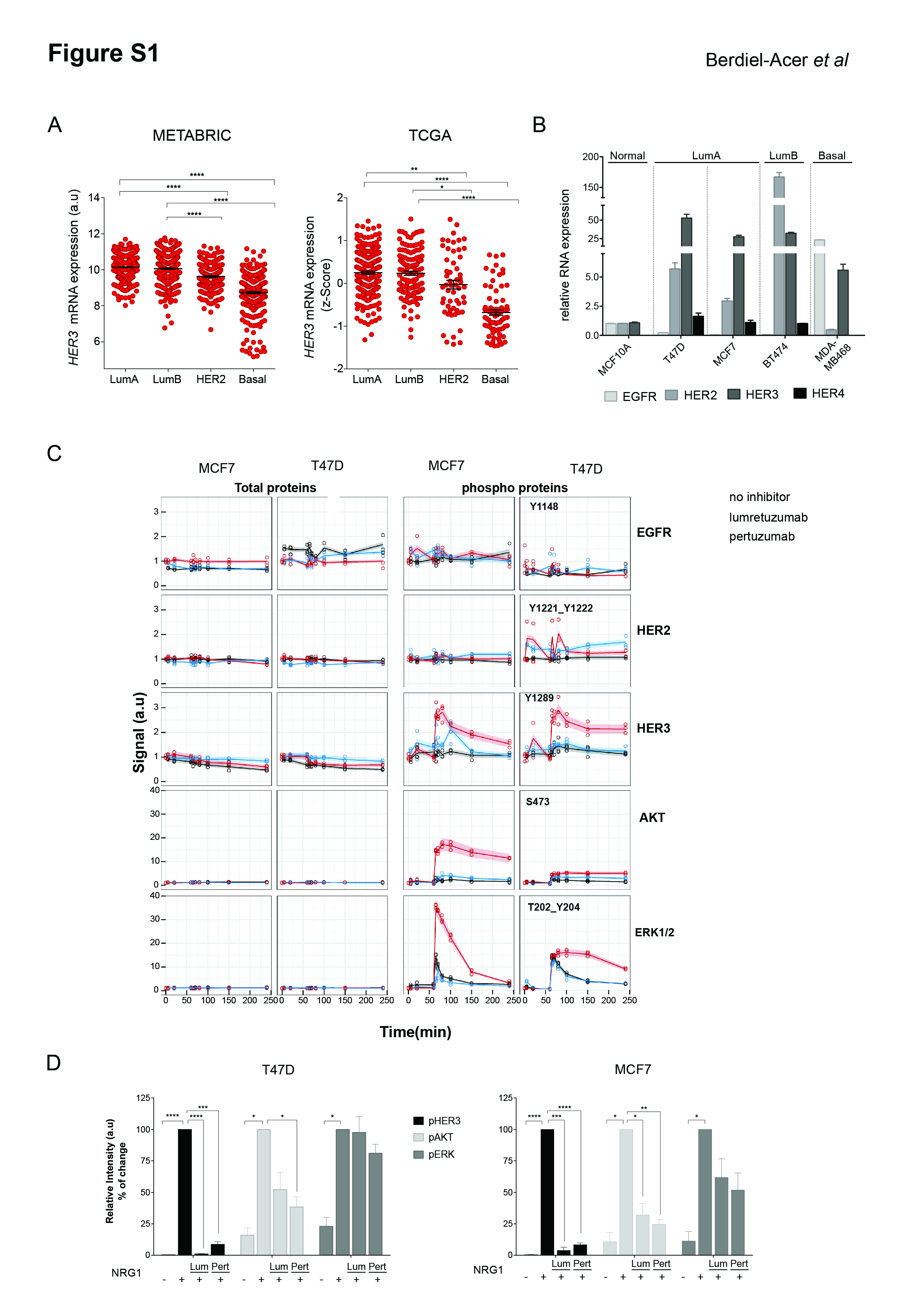

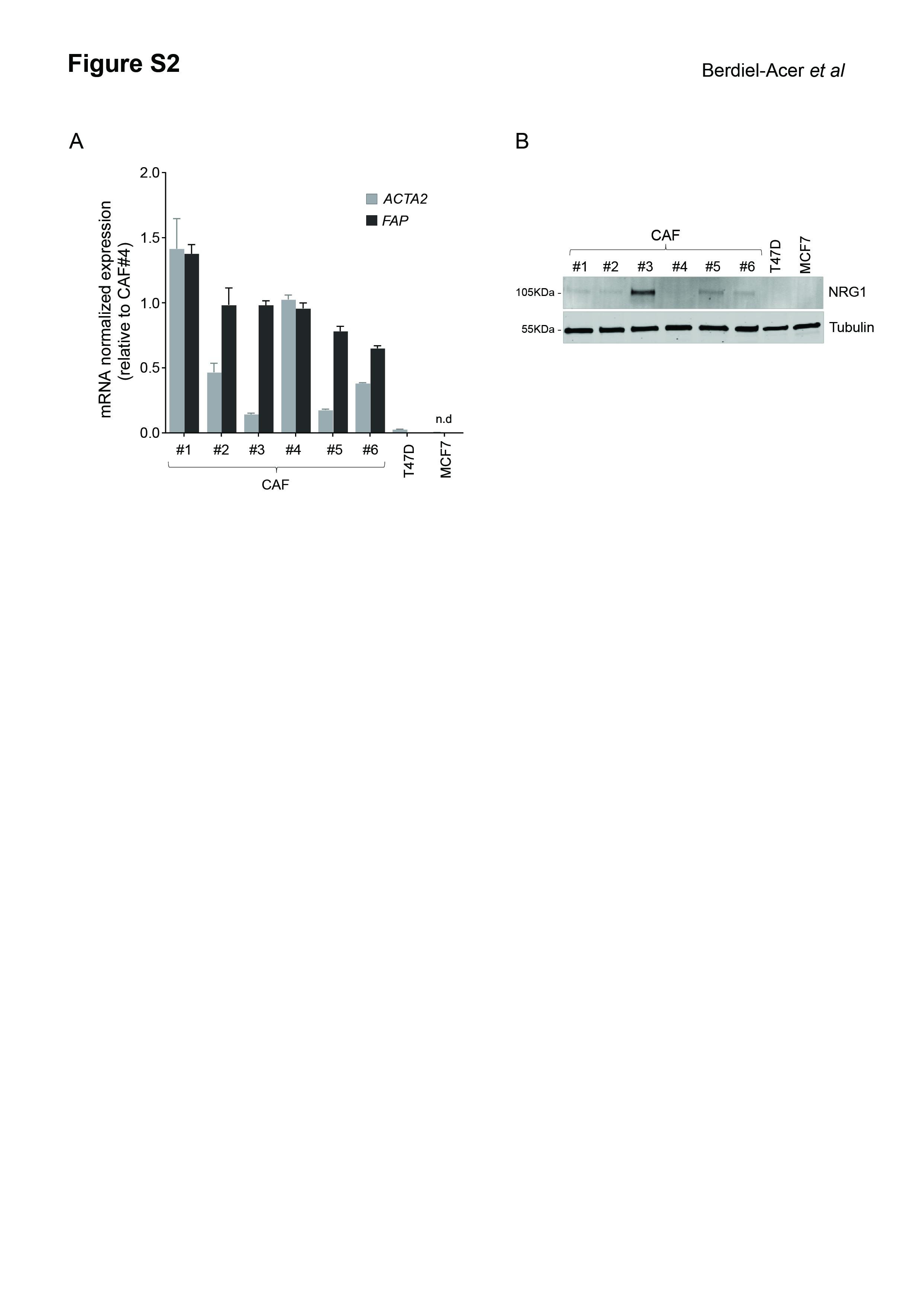

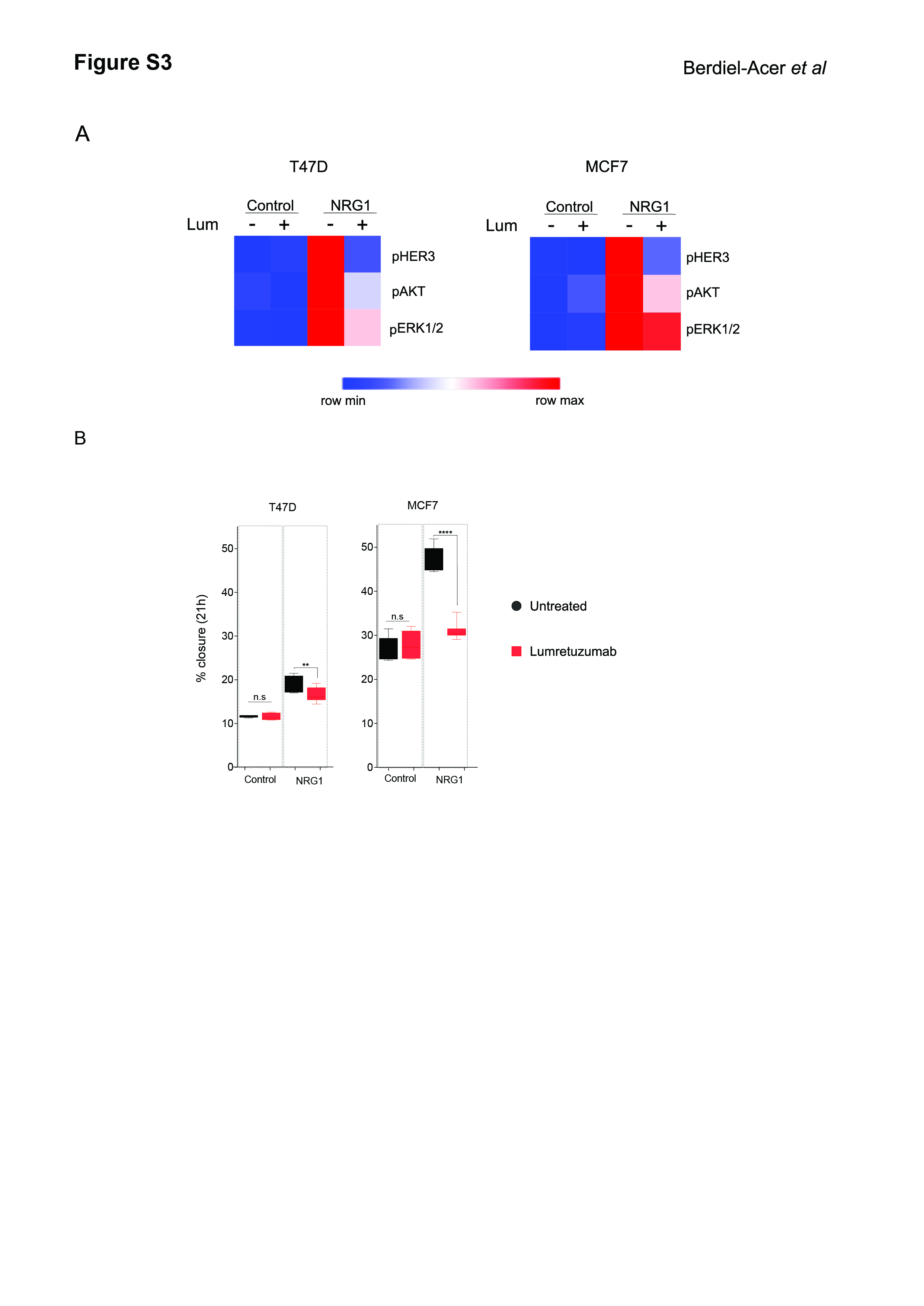

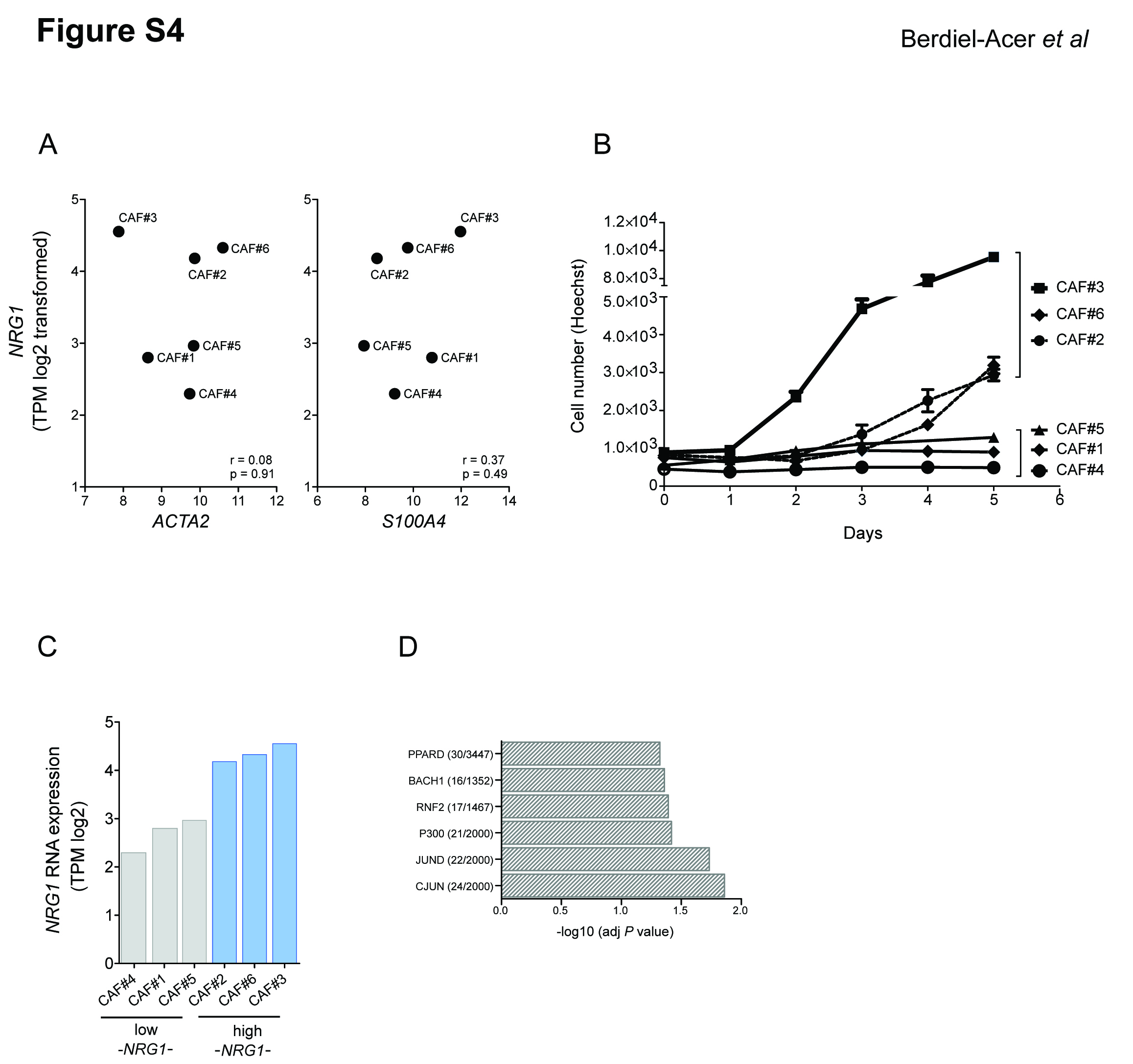

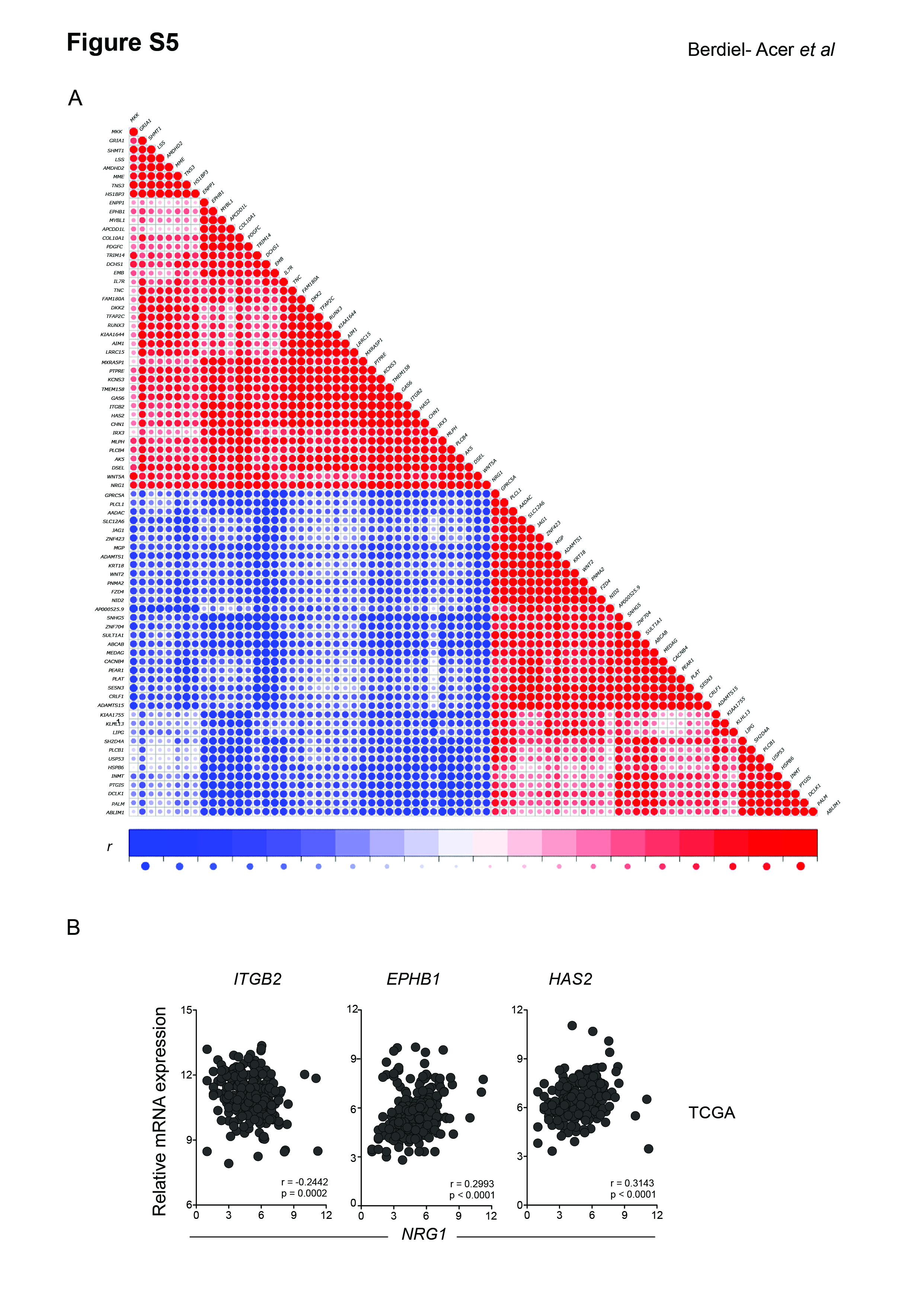

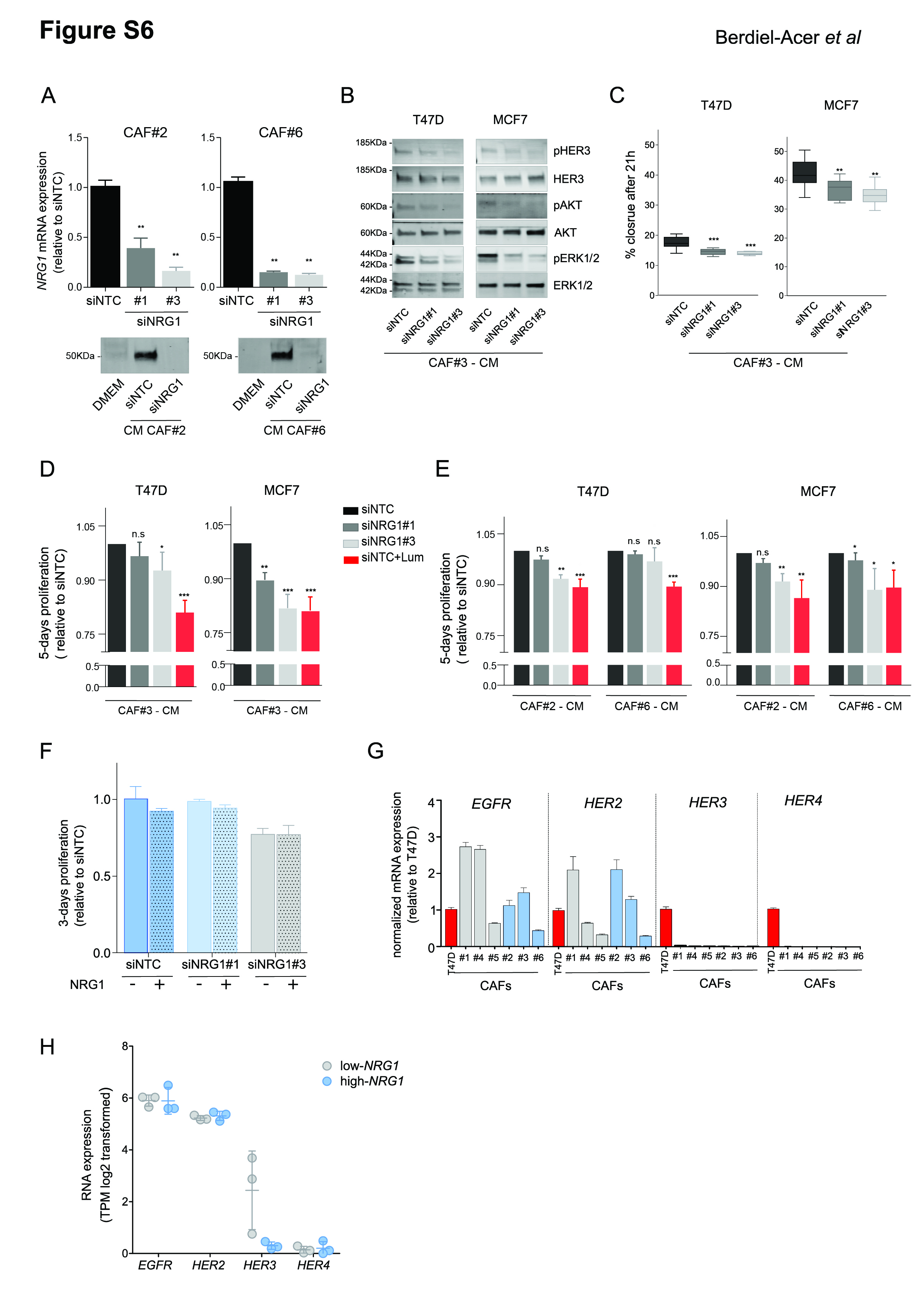


### Supplementary Figure Legends

**Figure S1.** ***HER3* expression in luminal subtypes and paracrine activation**

**A,** dot plots representing expression of *HER3* in breast cancer subtypes extracted from METABRIC and TCGA datasets. Mean +/- s.e.m represented. Statistically significant comparisons with luminal subtypes are depicted. ANOVA multiple comparison test ( **P* < 0.05, ***P* < 0.01,*****P* < 0.0001). **B,** mRNA expression of HER receptors in breast cancer cell lines representing different subtypes; T47D, MCF7 (luminal A), BT474 (luminal B), MDA-MB-468 (basal) breast cancer cell lines. Expression levels were normalized to the geometric mean of ACTB and GAPDH housekeeping genes expression. HER1-3 expression levels are shown relative to the respective levels in MCF10A, a non-transformed breast epithelial cell line. HER4 expression levels are shown relative to BT474. **C,** time course of protein intensities of EGFR, HER2, HER3, AKT, ERK1/2 and their respective phosphorylation states in T47D and MCF7 cancer cell lines treated with lumretuzumab (grey), pertuzumab (blue) or no inhibitor (red), obtained by reverse phase protein array (RPPA). Different phosphorylation dynamics are observed for MCF7 and T47D under treatment, mainly for ERK1/2. **D,** quantification of results shown in Fig 1E. Relative intensity shown as percentage of change in phosphorylation levels of HER3, AKT and ERK1/2 in T47D and MCF7 cancer cells under treatment with lumretuzumab or pertuzumab. Data is shown as mean +/- s.e.m of three independent replicates as in Fig1E. Only statistically significant comparisons with untreated condition are depicted. ANOVA multiple comparison test ( **P* < 0.05, ***P* < 0.01, ****P* < 0.001, *****P* < 0.0001).

**Figure S2. Characterization of carcinoma-associated fibrobasts (CAFs)**

**A,** mRNA values of α-smooth muscle actin (*ACTA2*) and fibroblast activation protein (*FAP*) in all fibroblasts under study and two luminal breast cancer cell lines (T47D and MCF7). Expression levels were normalized to the geometric mean of ACTB and GAPDH, and are shown relative to CAF#4. Bars represent mean +/- s.e.m of two independent experiments (n = 3 technical replicates each). **B,** representative western blot of full length NRG1 protein in all CAFs under study and in two luminal breast cancer cell lines. Tubulin was used as a loading control.

**Figure S3. Activation of HER3 in cancer cells by secreted NRG1 is CAF-dependent**

**A,** heatmap representing relative phosphorylation values of HER3, AKT and ERK1/2 in T47D and MCF7 quantified by RPPA. Low-serum condition media (1% FCS) was used as negative control and ectopic NRG-1β (50ng/ml) as a positive control. Controls correspond to the proteomic profile of T47D and MCF7 exposed to CM from CAFs with or without lumretuzumab in Fig 3A.

**B,** percentage of closure in a scratch assay.T47D and MCF7 cancer cells were cultured in DMEM-F12 1% FCS (with or without NRG-1β (50ng/ml)) and untreated (black) or treated with 10μg/ml lumretuzumab (red) during 21h. Closure is relative to untreated condition without NRG-1β. Box plots correspond to the mean and +/- s.e.m. of 2 independent experiments (n = 6 technical replicates). Two-tailed paired Student’s t-test ( ***P* <0.01, ****P* <0.0001).

**Figure S4. Heterogeneous expression of *NRG1* among CAFs**

**A,** correlation of *NRG1* and CAFs markers *ACTA2* and *S100A4* (FSP1) expression in the isolated CAFs based on TPM values. Spearman correlation *r* and *P* value are indicated.

**B,** number of CAFs quantified by nuclei staining (Hoechst) along 5 consecutive days in 10% FCS media. Each dot represents average of 2 independent replicates (n = 6 technical replicates each). **C,** ranking of fibroblasts based on *NRG1* expression (TPM - transcript per million) RNA seq data. CAFs were designated as low-*NRG1* (CAF#4, CAF#1 and CAF#5, grey) and high-*NRG1* (CAF#6, CAF#2 and CAF#3, blue) based on mean *NRG1* expression.**D,** graph bar representing adjusted *P* value and number of transcription factors in high-*NRG1* CAFs overlaping with the ChEA database.

**Figure S5. Genes correlating with *NRG1* expression**

**A,** correlation matrix of *NRG1* and associated genes. Matrix represents those genes with an absolute Pearson correlation > 0.8. **B,** correlation of *NRG1* and candidate genes in TCGA dataset samples. Only samples with tumour purity < 0.5 are represented. Spearman correlation r and *P* value are indicated per each gene/dataset.

**Figure S6. Downregulation of *NRG1* in CAFs**

**A,** (upper panel) downregulation of *NRG1* at RNA level in CAF#2 and CAF#6 using two independent siRNAs (siNRG1 #1, #3). Graph bars represent average of 3 independent experiments and 3 technical replicates each. U-Mann Whitney test comparing with siRNA non-targeting control (siNTC) ( ***P* < 0.01). **A,** (lower panel) Western blot showing immunoprecipitated NRG1 from the concentrated conditioned media of CAF#2 and CAF#6 treated with siRNA non-targeting control (siNTC) or siNRG1#3. Concentrated DMEM was used as a negative control. Images are representative of two independent experiments. **B,** protein levels of HER3, AKT, ERK1/2 and their phosphorylation status upon 5 min addition of CM from CAF#3 transfected with either siNRG1#1, siNRG1#3 or siRNA non-targeting control (siNTC). Western blot representative of 2 independent experiments. **C**, cancer cells and percentage of closure after 21h in CM from CAF#3 transfected with either siNRG1#1, siNRG1#3 or siNTC. Student’s t-test comparing individual siRNA NRG1 with siNTC ( ***P* < 0.01; ****P* < 0.001). **D**, graph bar representing proliferation of T47D and MCF7 cancer cells after 5 days with CM from CAF#3 transfected with either siNRG1#1, siNRG1#3 or siNTC (4 biological replicates, n=3 technical replicates each). Student’s t-test comparing individual siRNA NRG1 with siNTC ( **P* <0.05; ***P* < 0.01; ****P* < 0.001). **E,** graph bar representing proliferation of T47D and MCF7 cancer cells after 5 days with CM from CAF#2 or CAF#6 transfected with either siNRG1#1, siNRG1#3 or siNTC (4 biological replicates, n=3 technical replicates). Student’s t-test comparing individual siRNA NRG1 with siNTC (**P* <0.05; ***P* < 0.01; ****P* < 0.001). **F,** graph bar representing proliferation of CAF#3 line upon transfection with siRNA non-targeting control (siNTC), siNRG1#1 or siNRG1#3 without or with ectopic NRG-1β (50ng/ml) (doted bars). No significance rescue of proliferation was obtained when addition of ectopic NRG-1β in any of the conditions. **G**, normalized mRNA expression levels of HER receptors in CAF lines relative to expression of respective receptors in the T47D luminal cancer cell line obtained by RT-PCR. **H,** dot plot representing the TPM values of HER receptors in low-*NRG1* (grey circles) and high-*NRG1* (blue circles) CAFs obtained by RNA-sequencing.

### Legends for Supplementary Tables

**Table S1. Pathological characteristics of breast cancer specimens**

Selected histopathological details of the tumour samples. Staging of the tumours was done according to the TNM classification (1). Tumours were characterised based on positive (pos) or negative (neg) expression of biomarkers like estrogen receptor (ER), progesterone receptor (PgR) and HER2. ER expression was considered positive when > 1% of cells were stained. Percentage of cells staining positive for Ki67 is listed.

**Table S2. RNA seq expression CAFs vs. cancer cells**

TPM (transcript per million) expression values of 63 selected genes in 2 breast cancer cell lines (T47D and MCF7) and 6 CAFs lines. Color scale is shown per each line (blue= lowest value, red= highest value). Note: TPM numbers for this table were recalculated after obtaining sequencing results for the breast cancer cell line (see supplementary Methods).

**Table S3. List of most variable genes (MVG)**

A total of 517 genes were identified as statistically variable (*FDR* ≤ 0.001) among 6 CAFs under study.

**Table S4. Biological processes represented by MVG**

Gene Ontology of the most variable genes (MVG) reveals the biological processes in which those fibroblasts are contrasting. Processes related with locomotion, migration and cell motility are highly represented.

**Table S5. Transcription factors enriched in high-*NRG1* CAFs**

Enriched transcription factors and their adjusted p value in high-*NRG1* CAFs, obtained from the ChEA_2016 database.

**Table S6. List of differential expression genes between high and low-NRG1 CAFs**

Genes with and adj *P* value < 0.05 and absolute logFC > 0.5 are listed. Total of 102 genes were upregulated in the high-*NRG1* group and 151 genes upregulated in low-*NRG1*.

**Table S7. Correlation matrix of NRG1-associated genes**

Pearson correlation among the 517 most variable genes.

**Table S8. Materials and methods**

**A,** description of antibodies used for RPPA and Western blot.

**B,** sequences and probes for RNA expression analysis.

**C,** siRNA target sequence for *NRG1*. 2 independent siRNA were selected for each gene. A pool of 2 sequences of non-targeting siRNA used as a control.

### Supplementary Materials and Methods

**Cell culture**

The human MCF7 (HTB-22) and T47D (HTB-133) luminal breast cancer cell lines were obtained from the American Type Culture Collection (ATCC, LGC Standards GmbH, Wesel, Germany) and maintained in Dulbecco's Modified Eagle Medium: Nutrient Mixture F-12 (DMEM/F12), supplemented with 10% FCS, 50 units/ml penicillin and 50 µg/ml streptomycin sulfate (Invitrogen AG, Carlsbad, CA, USA) at 37°C with 5% CO_2_. The cell lines were authenticated by Multiplexion (Heidelberg, Germany) and negatively tested for mycoplasma contamination before and after completion of the study.

**RNA isolation and analysis**

Total RNA of primary CAFs and cancer cells was isolated with RNeasy Mini kit (Qiagen, Hilden, Germany) according to the manufacturer’s instructions. For mRNA, cDNA synthesis was carried out with the Revert Aid H Minus First Strand cDNA Synthesis Kit (Fermentas, Waltham, MA, USA). Quantitative RT-PCR (qRT-PCR) reactions for target genes were performed with the Applied Biosystems QuantStudio™ 3 & 5 Real-Time PCR System, using probes from the Universal Probe Library, UPL (Roche). The geometric mean of the housekeeping genes *ACTB* and *GAPDH* was used for normalization of mRNA analysis. List of primers are provided in Table S8.

**RNAseq comparison analysis CAFs and cancer cells**

Starting from the fastq files provided by the sequencer, reads were mapped to the human genome 38 using STAR, version 2.3 (2) and read counts per gene were determined using HTSeq-count, version 0.6.0 (3) and gencode annotations (release 34). TPMs (Transcript Per kilobase Million reads) were calculated in R (version 4.02, R Core Team 2020) using the Genomics features package to calculate exon sizes.

**Antibodies and immunoblotting**

For Western blotting, cells were lysed in ice-cold RIPA lysis buffer (Thermo Fisher Scientific) containing protease inhibitor Complete Mini and phosphatase inhibitor PhosSTOP (Roche). Protein concentrations were determined by BCA Protein Assay Reagent Kit (Thermo Fisher Scientific) and proteins were denatured with 4xRoti Load (Carl Roth, Karlsruhe, Germany) at 95°C for 5 min. Depending on the size, proteins were separated by 12 or 15% SDS-PAGE, blotted onto a PVDF membrane Immobilon-FL (Merck Millipore, Darmstadt, Germany) and incubated with primary antibodies overnight at 4°C. List of antibodies is provided in Table S8. Secondary IRDye®680 or IRDye®800-conjugated antibodies (LI-COR, Lincoln, NE, USA) were used for band visualization. Membranes were scanned and analyzed with Odyssey scanner and Odyssey 2.1, respectively (LI-COR, Lincoln, NE, USA). For quantification, local background subtraction and GAPDH/Tubulin normalization were applied.

**Immunofluorescence**

For detection of specific markers, 7.5x10^4^ fibroblasts were seeded in 6-well plate containing glass coverslips and cultured until 60-70% confluency. Cells were fixed with 4% paraformaldehyde for 10min at room temperature, permeabilized with 0.25% Triton X-100 for 10min and blocked with 3% BSA for 30min. Primary antibodies against α-smooth muscle actin (ab7817, 5µg/mL, Abcam) and Fibronectin (ab2413, 1:100, Abcam), were incubated overnight at 4ºC in a humidified chamber. 1h incubation with respective secondary antibodies containing DAPI (1:1000) was performed and images were acquired with Zeiss Cell Observer inverted microscope.

**ELISA (enzyme linked immunosorbent assay)**

Conditioned media was concentrated using Amicon®Ultra 3kDa-cutoff centrifugal units following (UFC900324,Merck Millipore). 1 hour centrifugation (4,000 g, 4°C) of 12ml CM allowed for the retrieval of ~300µl concentrate (40-fold concentration). NRG1 was detected with a DuoSet® Human NRG1-β/HRG-β1 ELISA kit (DY377-05, R&D Systems) following the manufacturer’s instructions. The optical density (450nm substracted by 560nm wavelength) was determined with a microplate reader (GloMax Discover, Promega) and a standard curve generated and used to calculate the NRG1 levels in the concentrated CM.

**NRG1 Immunoprecipitation**

8 x 10^6^ fibroblasts were seeded and their conditioned media collected for NRG1 pull-down. Briefly, the conditioned media was filter-concentrated through a cellulose membrane with 3kDa-cutoff (UFC900324 Amicon®Ultra devices, Merck Millipore). One-hour centrifugation (4,000 g, 4°C) allowed a 40-fold concentration. Per sample, 200 µg of magnetic beads (Dynabeads 280, ThermoFisher) were incubated for 45 min at RT with 2 µg of biotinylated anti-NRG1 antibody (BAF377, R&D) for conjugation. Afterwards, beads were washed 4 times with PBS + 1% BSA. The antibody-bead conjugate was then incubated overnight at 4ºC with the conditioned-media. Samples were washed two times with PBS + 0.01% Tween and a final time with PBS before elution. To elute the antibody-antigen conjugate from the streptavidin beads, RIPA buffer was added and samples were incubated for 5 min at 95ºC. Samples were loaded in a 12% SDS-page, blotted onto a PVDF membrane Immobilon-FL (Merck Millipore, Darmstadt, Germany) and incubated with primary antibody (MAB377, R&D) overnight at 4°C. Secondary IRDye®680-conjugated antibody (LI-COR, Lincoln, NE, USA) was used for band visualization. Membranes were scanned and analysed with Odyssey scanner and Odyssey 2.1, respectively (LI-COR, Lincoln, NE, USA).

**Viability and proliferation assays**

3000 cancer cells were seeded in 96-well white plates and the effect of the different treatments/ CM on cell viability was evaluated using CellTiter-Glo® luminescent assay (G7570, Promega). Prior to addition of CM or NRG-1β (50ng/ml), cells were pre-treated with 10μg/ml lumretuzumab or pertuzumab for 30min. Luminescence was determined after 3 days of culture using the GloMax® microplate reader (GM3000, Promega) and normalized with seeding control plate.

To determine proliferation rate, 1 000 fibroblasts or 3 000 cancer cells, were seeded in 96-well black plates and cell counting was measured by nuclei staining. Hoechst 1/10 000 (33342, Thermo Fisher), was added for 45 minutes prior to cell acquisition with MetaXpress microscope (Molecular Devices) at the desired time points.

**Migration assays**

For scratch assay, 30,000 MCF7/T47D cells (in DMEM-F12 10% FCS, 1% P/S ) were seeded in 96-well black plates. Cells were allowed to grow until confluence, and starved O/N in 1% FCS media. The following day, cells were stained with Cell Tracker™ Green CMFDA (Invitrogen C2925, 10mM in DMSO) at a 1:5,000 dilution in starvation media for 30min at 37⁰C. Afterwards, media was replaced by fresh starvation media and incubated for additional 30-45min at 37⁰C to allow for the metabolization of the dye. By using a 96-well multichannel pipettor, a scratch was performed in each well. Wells were washed with PBS several times to remove any floating cell and 100μl/well of the corresponding conditions in 1% FCS were added. Images were acquired at the initial time point and after 21h with MetaXpress microscope (Molecular Devices). Images were analyzed using the MRI Wound Healing Tool in ImageJ (5), and the gap closure was determined by comparing the scratch surface areas at 0h and 21h post CM addition.

For migration of fibroblasts, 30,000 CAFs were seeded in the upper chamber of a Transwell plate (Corning® 3422, 8μm pore size) in media without FCS. Media containing 10% FCS was added to the bottom well. After 8h, cells were fixed with 4% PFA and stained with crystal violet (CV). Images were taken for the different conditions and elution of the crystal violet with acetic acid was used to quantify absorbance. Normalization was done by eluting the CV of the same number of cells seeded in an independent well.

**Transfections**

Transfections with siRNA were performed using Lipofectamine RNAimax (Invitrogen) according to the manufacturer’s instructions. ON TARGETplus siRNAs were obtained from Dharmacon (Lafayette, CO, USA). For each gene, individual siRNAs were tested and two were selected for further experiments. ON TARGETplus nontargeting siRNA pool (Dharmacon) was used as control. For the siRNA transfection, fibroblasts were seeded in 10cm dishes with their growth medium without antibiotics. Twenty-four hours later, cells were transfected with siRNA at a final concentration of 20nM. Twenty-four hours after transfection, fibroblasts were re-seeded for the different assays. Sequences of siRNA used are in Table S8.

**Transcription Factor enrichment analysis**

The transcription factor enrichment analysis was performed using the ChEA_2016 database (6) with the Enrichr gene list enrichment analysis tool (7, 8) and selecting the significantly enriched subset of transcription factors that included NRG1 in their targets.

**Differential expression analyses**

Differential expression analyses between low NRG1 expressing CAF lines (CAF#1,#4 and #5) and high NRG1 expressing CAF samples (CAF#2,#3 and #6) were performed using DESeq2 (version 1.30.0) (9, 10). Correction for multiple testing was performed using Benjamini & Hochberg's adjustment of p-values (11). Genes were considered significantly differentially expressed if adj P value < 0.05 and absolute logFC > 0.5.

**Analysis of expression datasets (LCM)**

For the analysis of the GEO datasets with the accession numbers: GSE10797 (12), GSE14548 (13), GSE35019 (14) and GSE83591 (15), normalized data were downloaded from GEO.

**Identification of highly variable genes**

In order to identify the most variable genes in the CAF lines, the deviation of gene expression levels from a fitted regression line estimated on coefficient of variation of control feature expressions were obtained using generalized linear models (16, 17). To control the effects of outlier in the data, a winsorization procedure was used on the expression matrix. 517 highly variable genes were identified via Χ^2^ test at *FDR* ≤ 0.001.

**Statistical analyses**

Statistical analyses and graphical representation were performed using GraphPad Prism version 6.00 for Windows.

### Supplemental References

1. Sobin LH, Gospodarowicz, M. K., and Wittekind, C. TNM classification of malignant tumours: John Wiley & Sons.; 2011.

2. Dobin A, Davis CA, Schlesinger F, Drenkow J, Zaleski C, Jha S, et al. STAR: ultrafast universal RNA-seq aligner. Bioinformatics. 2013;29(1):15-21.

3. Anders S, Pyl PT, Huber W. HTSeq--a Python framework to work with high-throughput sequencing data. Bioinformatics. 2015;31(2):166-9.

4. Lawrence M, Huber W, Pages H, Aboyoun P, Carlson M, Gentleman R, et al. Software for computing and annotating genomic ranges. PLoS Comput Biol. 2013;9(8):e1003118.

5. Schindelin J, Arganda-Carreras I, Frise E, Kaynig V, Longair M, Pietzsch T, et al. Fiji: an open-source platform for biological-image analysis. Nat Methods. 2012;9(7):676-82.

6. Lachmann A, Xu H, Krishnan J, Berger SI, Mazloom AR, Ma'ayan A. ChEA: transcription factor regulation inferred from integrating genome-wide ChIP-X experiments. Bioinformatics. 2010;26(19):2438-44.

7. Chen EY, Tan CM, Kou Y, Duan Q, Wang Z, Meirelles GV, et al. Enrichr: interactive and collaborative HTML5 gene list enrichment analysis tool. BMC Bioinformatics. 2013;14:128.

8. Kuleshov MV, Jones MR, Rouillard AD, Fernandez NF, Duan Q, Wang Z, et al. Enrichr: a comprehensive gene set enrichment analysis web server 2016 update. Nucleic Acids Res. 2016;44(W1):W90-7.

9. Anders S, Huber W. Differential expression analysis for sequence count data. Genome Biol. 2010;11(10):R106.

10. Love MI, Huber W, Anders S. Moderated estimation of fold change and dispersion for RNA-seq data with DESeq2. Genome Biol. 2014;15(12):550.

11. Y.Hochberg YBa. Controlling the False Discovery Rate: A Practical and Powerful Approach to Multiple Testing. Journal of the Royal Statistical Society: Series B (Methodological). 1995;57(1).

12. Casey T, Bond J, Tighe S, Hunter T, Lintault L, Patel O, et al. Molecular signatures suggest a major role for stromal cells in development of invasive breast cancer. Breast Cancer Res Treat. 2009;114(1):47-62.

13. Ma XJ, Dahiya S, Richardson E, Erlander M, Sgroi DC. Gene expression profiling of the tumor microenvironment during breast cancer progression. Breast Cancer Res. 2009;11(1):R7.

14. Vargas AC, McCart Reed AE, Waddell N, Lane A, Reid LE, Smart CE, et al. Gene expression profiling of tumour epithelial and stromal compartments during breast cancer progression. Breast Cancer Res Treat. 2012;135(1):153-65.

15. Liu H, Dowdle JA, Khurshid S, Sullivan NJ, Bertos N, Rambani K, et al. Discovery of Stromal Regulatory Networks that Suppress Ras-Sensitized Epithelial Cell Proliferation. Dev Cell. 2017;41(4):392-407 e6.

16. Trevor J. Hastie DP. Generalized Linear Models. In: Hastie TJ, editor. Statistical Models in S. New York2017. p. 53.

17. Nelder PMaJA. Generalized Linear Models Taylor & Francis Group; 1989.
