# Supplementary figures and images for: Stromal NRG1 in luminal breast cancer defines pro-fibrotic and migratory cancer-associated fibroblasts
Source: Oncogene. 2021 Mar 10;40(15):2651–66. doi: 10.1038/s41388-021-01719-3 (PMC8049869; doi:10.1038/s41388-021-01719-3)

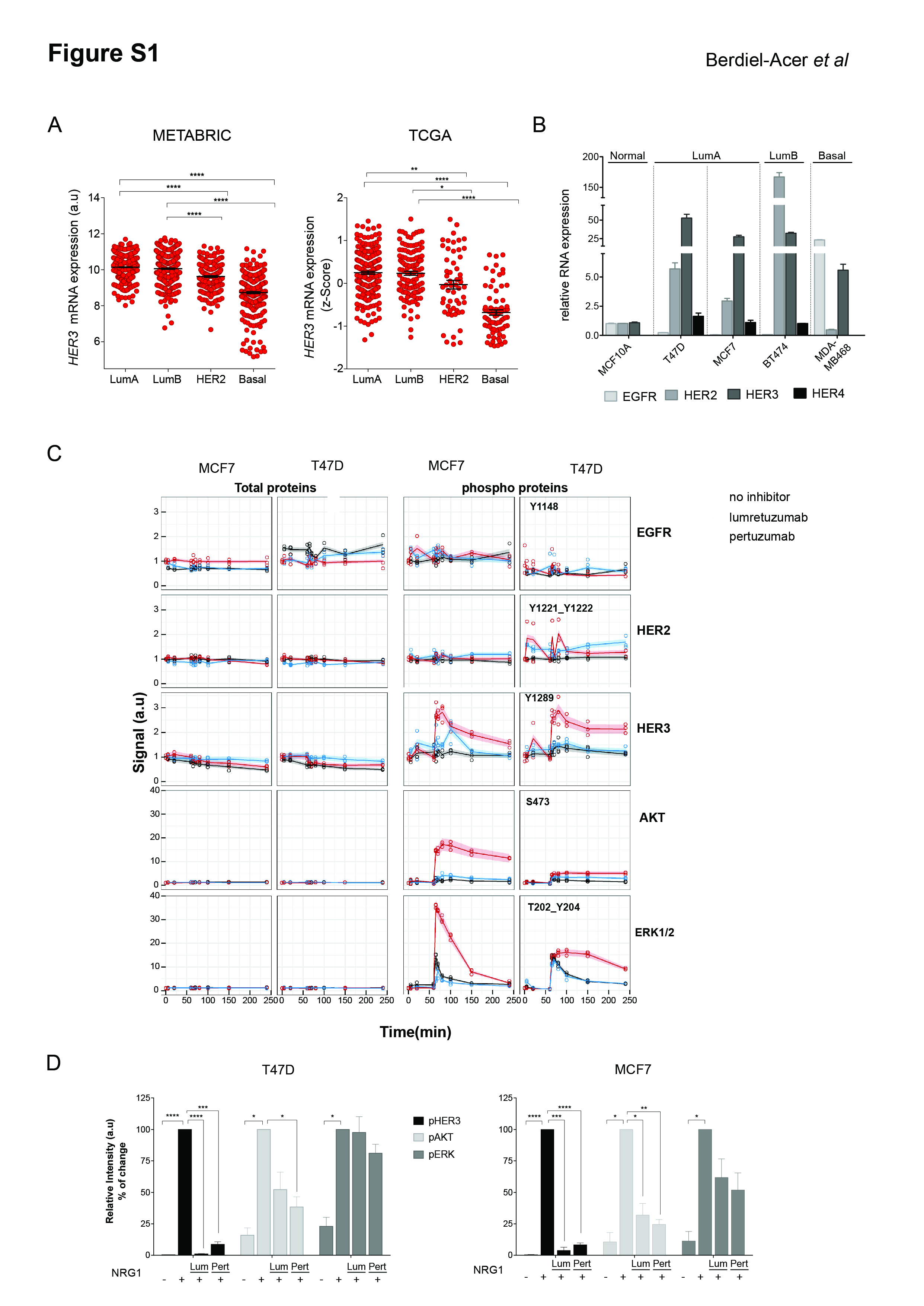

Supplement: Supplementary file 2 — Figure S1 [file 41388_2021_1719_MOESM2_ESM.jpg]

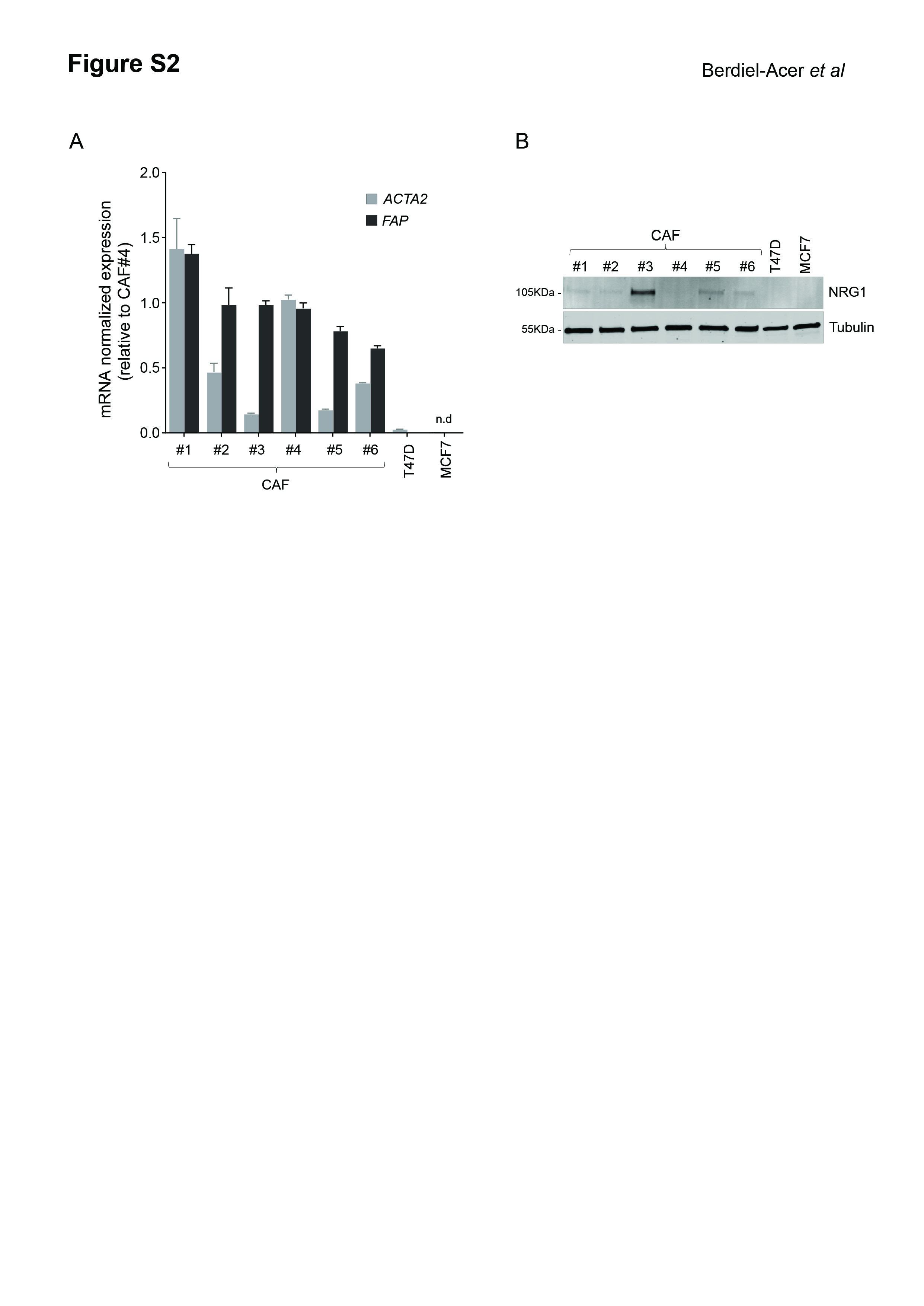

Supplement: Supplementary file 3 — Figure S2 [file 41388_2021_1719_MOESM3_ESM.jpg]

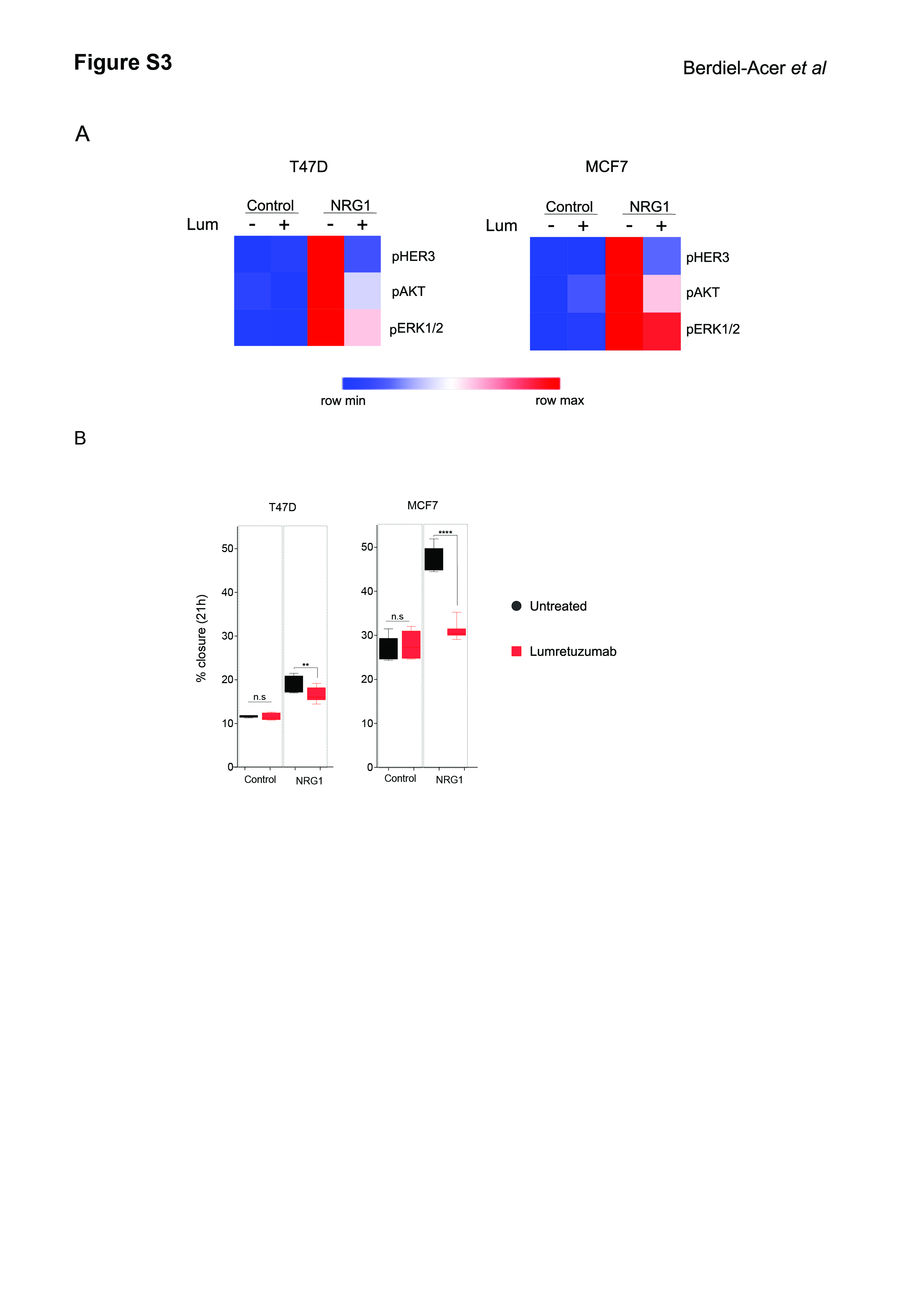

Supplement: Supplementary file 4 — Figure S3 [file 41388_2021_1719_MOESM4_ESM.jpg]

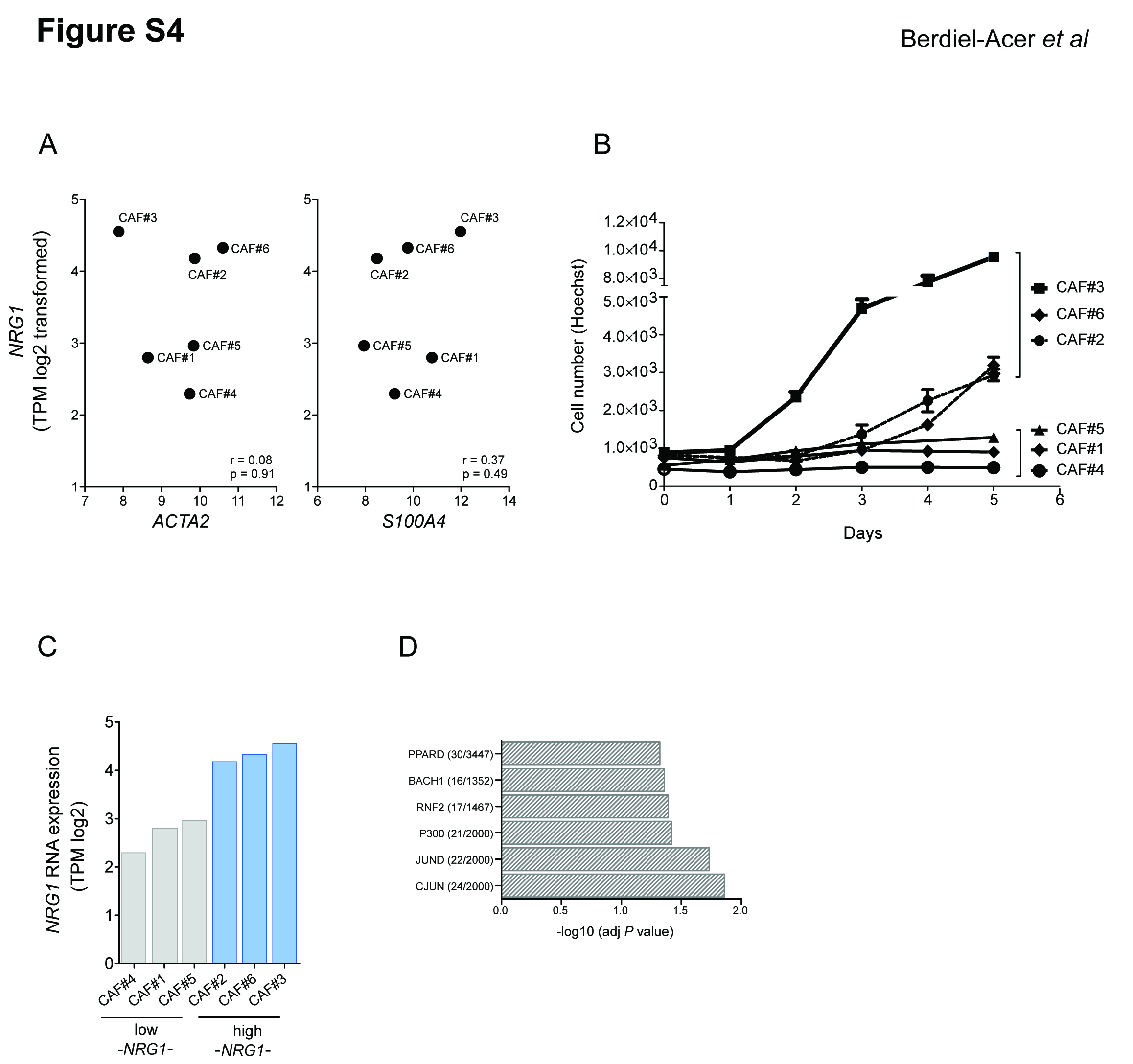

Supplement: Supplementary file 5 — Figure S4 [file 41388_2021_1719_MOESM5_ESM.jpg]

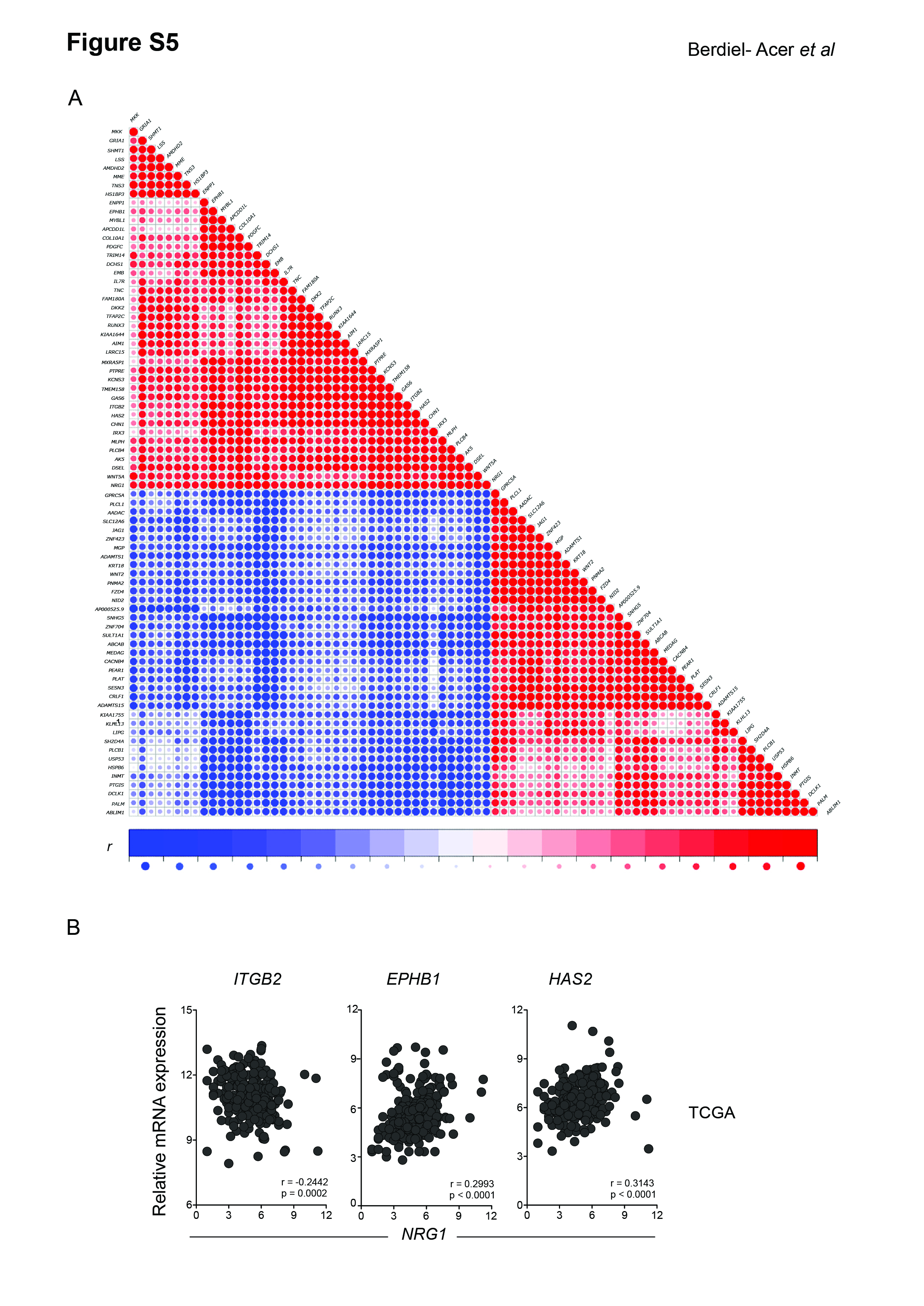

Supplement: Supplementary file 6 — Figure S5 [file 41388_2021_1719_MOESM6_ESM.jpg]

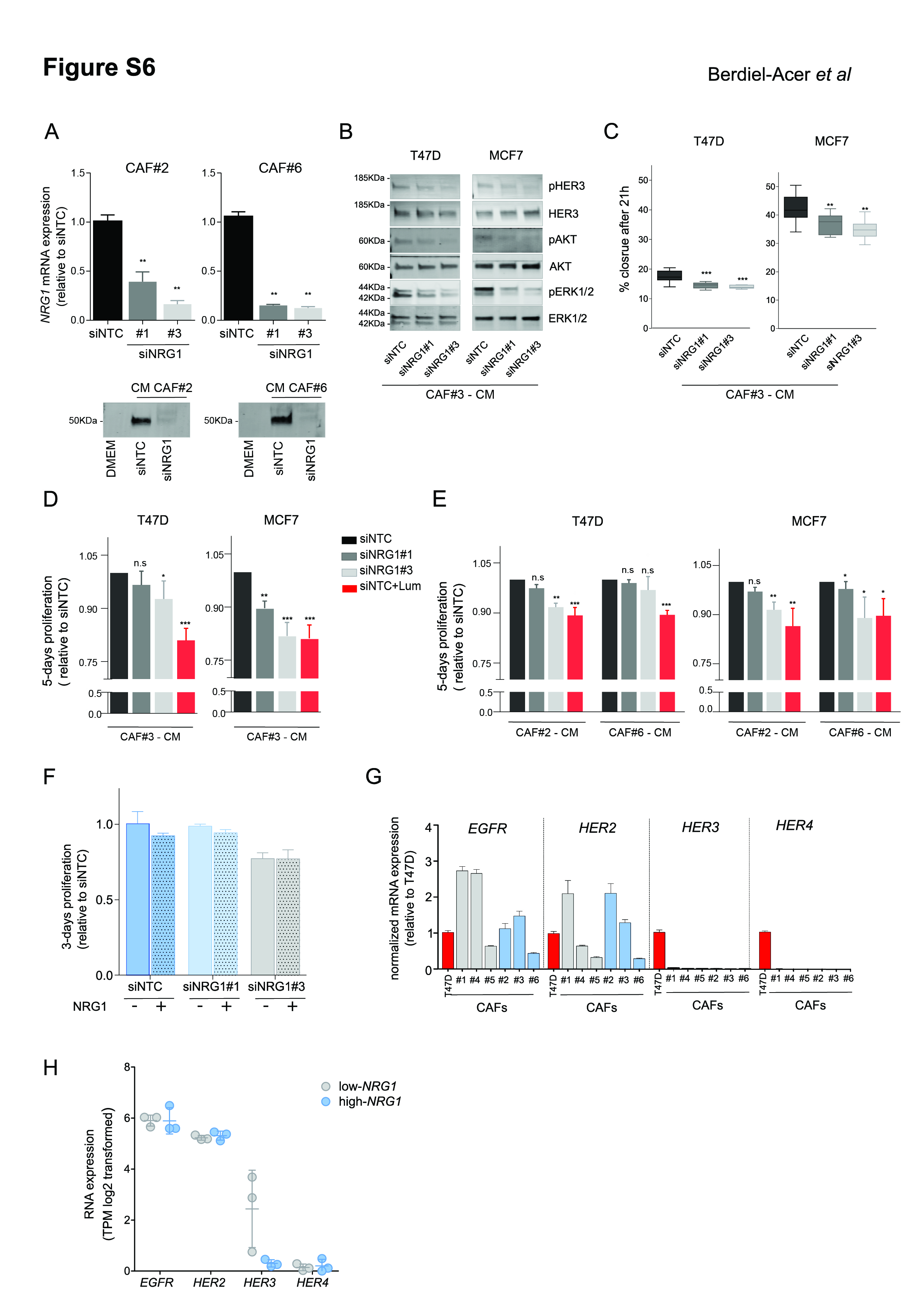

Supplement: Supplementary file 7 — Figure S6 [file 41388_2021_1719_MOESM7_ESM.jpg]
